# Colitis Is Effectively Ameliorated by (±)-8-Acetonyl-dihydrocoptisine via the XBP1-NF-κB Pathway

**DOI:** 10.3389/fphar.2017.00619

**Published:** 2017-09-05

**Authors:** HaiJing Zhang, GuangMing Song, ZhiHui Zhang, HuaChen Song, XiaoNan Tang, AnJun Deng, WenJie Wang, LianQiu Wu, HaiLin Qin

**Affiliations:** ^1^State Key Laboratory of Bioactive Substances and Functions of Natural Medicines, Institute of Materia Medica, Chinese Academy of Medical Sciences and Peking Union Medical College Beijing, China; ^2^Department of Pharmacology, Logistics University of PAPF Tianjin, China

**Keywords:** ulcerative colitis, X-box binding protein 1, NF-κB, cytokines, coptisine

## Abstract

Ulcerative colitis (UC) is a recurrent, chronic intestinal disease. Available treatments for UC are poor effective and/or cause severe adverse events. X-box binding protein 1 (XBP1) and nuclear factor-κB (NF-κB) have been reported to play important roles in UC. Specifically, deletion or downregulation of XBP1 leads to spontaneous enteritis and results in imbalanced secretion of NF-κB and other proinflammatory cytokines. (±)-8-acetonyl-dihydrocoptisine, i.e., (±)-8-ADC, is a monomer semi-synthesized from coptisine. *In vitro*, (±)-8-ADC activated the transcriptional activity of XBP1, inhibited expression of NF-κB, and reduced production of proinflammatory cytokines, such as tumor necrosis factor alpha (TNF-α) and interleukin-1 beta (IL-1β), in lipopolysaccharide-stimulated IEC6 cells. Therefore, silencing XBP1 would reduce the inhibition effect of (±)-8-ADC on NF-κB expression and the cytokines secretion *in vitro*. In a dextran sulfate sodium-induced colitis mouse model, oral administration of (±)-8-ADC ameliorated weight loss and colon contracture, and decreased the average disease activity index score and pathological damage. Simultaneously, (±)-8-ADC also increased XBP1 expression, and decreased NF-κB expression and secretion of myeloperoxidase, TNF-α, IL-6 and IL-1β in the colon. Therefore, (±)-8-ADC may ameliorate UC via the XBP1-NF-κB pathway and should be considered as a therapeutic candidate for UC.

## Introduction

Ulcerative colitis (UC) is a recurrent, debilitating, and chronic inflammatory disease of the colon and rectum that is characterized by mucosal and submucosal ulceration, diarrhea, and rectal bleeding (Akiho et al., 2015). These symptoms can severely impact patient quality of life. In addition, the combined incidence of UC in all centers worldwide has been rising by about 0.01%, with a prevalence of up to 0.5% in Western populations (Novak et al., 2017). Furthermore, the risk of colitis-associated colon carcinogenesis increases with progression of UC.

A number of therapeutic agents have been developed to treat UC, including aminosalicylic acids, (e.g., sulfanilamide pyridine [SASP]), antibiotics (e.g., penicillin and chloramphenicol), oral corticosteroids, and immunosuppressive agents (e.g., azathioprine). However, these treatments have been ineffective and are associated with severe adverse events, including gastrointestinal adverse reactions, headaches, hemolysis, and allergies (Peppercorn, 1984). Recently, new families of biological agents aimed at treating UC have been developed, including tumor necrosis factor-α (TNF-α) inhibitors. Adalimumab and infliximab are both anti-TNF agents that have been approved for the treatment of UC. However, these anti-TNF agents have some considerable disadvantages, such as being expensive, increasing risk of infection, and having a risk of secondary failure due to intolerance following long-term use (Rogler, 2015; Novak et al., 2017). Therefore, there is still a need to develop new therapeutic agents against UC with low toxicity and minimal side effects.

Ulcerative colitis pathogenesis has yet to be fully delineated. Several studies have reported that immune dysfunction, as well as XBP1, plays an important role in the development UC (Pedersen et al., 2014; Sands, 2014). XBP1 is genetically associated with UC, where deletion of XBP1 affects the intestinal epithelium and results in spontaneous intestinal inflammation (Adolph et al., 2013) and XBP1^ΔIEC^ mice have aggravated dextran sodium sulfate (DSS)-induced colitis compared to wild-type mice (Kaser et al., 2008). Genetic XBP1 defects can lead to spontaneous enteritis and imbalanced production of cytokines, including NF-κB and other proinflammatory cytokines (Li et al., 2011). Interestingly, the NF-κB signaling pathway is regarded as one of the key pathways responsible for UC (Bank et al., 2014). NF-κB can induce the expression of a large array of inflammatory mediators, including TNF-α, which is one of the pro-inflammatory cytokine, along with IL-1β and IL-6, reported to play central roles in modulating inflammation during UC.

**FIGURE 1:**
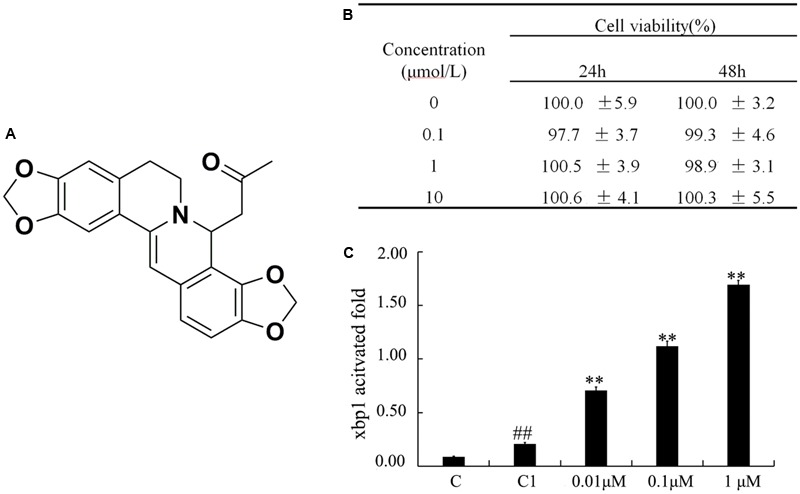
Cytotoxicity of (±)-8-ADC on IEC6 cells and effect on transcriptional activation of XBP1. **(A)** The chemical structure of (±)-8-ADC and its patent number (CN104211709A). Patent details can be found in the Supporting Information. **(B)** IEC6 cell cytotoxicity from (±)-8-ADC measured using the CCK8 assay (*n* = 3). **(C)** Effect of (±)-8-ADC on XBP1 transcriptional activation. C refers to the background contrast and C1 to the pGL3-basic vector control. ^##^*p* < 0.01 compared with the C group; ^∗∗^*p* < 0.01 compared with the C1 group.

Purification of (±)-8-ADC monomer was first reported by Zhang et al. (2015) and we have since synthesized it in large quantities. The chemical structure of (±)-8-ADC is depicted in **Figure 1A**. (±)-8-ADC is a derivative of coptisine, which has anti-inflammatory effects that have been previously reported. Lee et al. (2012) reported that coptisine inhibits NF-κB p65 phosphorylation via the RANK signaling pathway. In addition, Zou et al. (2015) determined coptisine is a good candidate for preventing obesity-related diseases by acting through the LPS/Toll-like receptor-4-mediated signaling pathway. Fan et al. (2014) described a Chinese medicinal herb recipe commonly prescribed for the treatment of UC containing coptis. Luo et al. (2010) found that combinations of crude rhizomes, including coptis, could modulate mucosal immune responses in DSS-induced mice. However, the above groups only evaluated combinations containing coptis. Therefore, to our knowledge, this is the first detailed report demonstrating that the compound monomer (±)-8-ADC is highly effective at ameliorating experimental colitis and has no adverse effects in healthy mice. Furthermore, the anti-UC activity of (±)-8-ADC was superior to that of coptisine (see Supporting Information).

The DSS-induced colitis mouse model is currently a commonly used model in UC research and it resembles human UC in terms of histology and pathological process (Ren et al., 2015). This present study was undertaken to investigate the mechanism of action of (±)-8-ADC prevention of the development of colonic inflammation, and it was found to act via the XBP1-NF-κB pathway in a DSS-induced colitis mouse model and the IEC6 cell line.

## Materials and Methods

### Synthesis of (±)-8-ADC

(±)-8-ADC (99.5% purity) was synthesized by Zhang et al. (2015) at the Institute of Materia Medica of the Chinese Academy of Medical Sciences. Quaternary coptisine was isolated previously from certain *Coptis* and *Corydalis* species. Acetone (14.10 mmol) was added dropwise to a solution of quaternary coptisine (2.81 mmol) stirred into 5 N NaOH (7 mL). After stirring for 1 h at room temperature, the reaction mixture was filtered, washed with water (100 mL), and then recrystallized from acetone to generate (±)-8-ADC (740 mg, 69.8% yield), which is a yellow crystal.

### Reagents

All synthesized reagents and solvents were either reagent grade or purified using standard methods prior to use. Anhydrous solvents and reagents were all of analytical purity and dried using routine protocols.

DSS (w/v; MW36000-50000) was purchased from MP Biomedicals (Santa Ana, CA, United States), fetal bovine serum (FBS) from GIBCO (Grand Island, NY, United States), lipopolysaccharide (LPS) from Sigma Chemical Co. (St. Louis, MO, United States), and bicinchoninic acid (BCA) assay kits from Beyotime Biotechnology (Beijing, China). ELISA kits for TNF-α, IL-1β, and IL-6 were purchased from R&D Systems, Inc. (Minneapolis, MN, United States), the myeloperoxidase (MPO) (A044) and fecal occult blood test kits (C027) from Nanjing Jiancheng Bioengineering Institute (Nanjing, China), and the RNAsimple Total RNA and Quant One Step RT-qPCR Kits (SYBR Green) were from TIANGEN BIOTECH (Beijing, China). Phospho-NF-κB p65, XBP1, and β-actin specific antibodies were obtained from Cell Signaling Technology, Inc. (California, United States). The TransDetect Cell Counting Kit-8 (CCK-8) and TransDetect Double-Luciferase Reporter Assay Kits were purchased from TransGen Biotech (Beijing, China).

### Cell Culture

IEC6 cells (ATCC) were cultured in RPMI 1640 medium supplemented with 10% FBS for cytotoxicity, and XBP1 reporter gene assays, and subsequent Western-blot. Cell supernatants were collected for the pro-inflammatory cytokine assays measuring for TNF-α and IL-1β, and ELISAs were performed according to the kit instructions.

### Cytotoxicity Assays

(±)-8-ADC was added to 96-well plate containing 5 × 10^4^ cells/well of IEC6 cells in the growth phase and the plates were incubated at 37°C for 24 or 48 h with 5% CO_2_. CCK reagent (20 μL) was then added into each well and the absorbance was measured at 450 nm.

### XBP1 Gene Expression Reporter Assay

The effect of (±)-8-ADC on the transcription of XBP1 in IEC6 cells was determined. IEC6 cells were transiently transfected with a XBP1 expression plasmid and the pRL-CMV-Renilla plasmid was co-transfected as a control. After transfection, the cells were pretreated with (±)-8-ADC (0.01, 0.1, or 1 μM) for 24 h. A luciferase assay was performed using the TransDetect^®^ Double-Luciferase Reporter Assay Kit according to the manufacturer’s instructions.

### SiXBP1 Synthesis and Transfection

SiXBP1 (5′-CAAGCUGGAAGCCAUUAAUTT-3′) was synthesized by GenePharma. IEC6 cells were plated in a 6-well plate and then wells were divided randomly into six groups. Three groups were transfected with the siXBP1(-) negative control: the control, LPS (5 μg/mL) stimulated, and (±)-8-ADC (1 μM) groups. Meanwhile, three group were transfected with siXBP1, i.e., siXBP1(+) groups: control, LPS (5 μg/mL) stimulated, and (±)-8-ADC(1 μM) groups. Cells were seeded in 6-well plates and grown until 70–80% confluent. IEC6 cells were then transiently transfected with siRNA using Lipofectamine^TM^ 2000 according to the manufacturer’s instructions and then incubated in serum starved media at 37°C in 5% CO_2_. At 4 h post-transfection, the media was replaced with fresh complete medium containing 10% FBS. The cells were harvested 24 h after transfection to be analyzed by Western-blot.

### Animals

Male C57BL/6J mice (6 weeks, 18–22 g) were obtained from Vital River Laboratories (Beijing, China). Prior to experimentation, the animals were kept at 24 ± 1°C at 50–60% humidity with a 12-h light-dark cycle starting at 8:00 AM for 1 week, and provided with standard pellet chow and water *ad libitum*.

Experiments were performed in accordance with the Guide for the Care and Use of Laboratory Animals approved by the Experimental Animal Research Center of the Academy of Medical Sciences, China, as well as relevant guidelines and regulations of the Chinese Academy of Medical Science (CAMS). Experimental procedures were approved by the Institutional Ethical Committee for Animal Care and Use of CAMS. After the experiments were completed, the animals were anesthetized with pentobarbital (80 mg/kg) and euthanized.

### DSS-Induced Acute UC in Mice and (±)-8-ADC Treatment

C57BL/6J mice were randomly divided into 7 groups of 6 mice each: control (normal water), vehicle (300 mg/kg (±)-8-ADC), DSS-induced (2.3% DSS in drinking water), SASP (300 mg/kg), and three (±)-8-ADC (75, 150, and 300 mg/kg) groups. The vehicle group was received 300 mg/kg (±)-8-ADC but was not treated with DSS. Except for the control and the vehicle groups, colitis was induced in all mice by orally administering 2.3% DSS for 7 consecutive days. The SASP group was treated with 300 mg/kg SASP, and the (±)-8-ADC groups received 75, 150, or 300 mg/kg (±)-8-ADC orally from the start until the end of the experiment. The DAI score was determined over the course of 7 days as previously described (Kaser et al., 2008), and was as follows: weight loss (%) (0: normal, 1:1–5%, 2:5–10%, 3:10–15%, and 4:>15%), stool consistency (0 and 1: normal, 2 and 3: loose stool, and 4: diarrhea), and stool blood (0: negative, 1:±, 2:+, 3:++, and 4: gross). After the 7-day experimental period, the mice were sacrificed. The fecal occult blood test was performed as described by the manufacturer’s instructions. The colons of the mice were excised, opened longitudinally, and rinsed with an ice-cold saline solution. The excised colons were then divided longitudinally into two parts, where one part was immediately fixed in 10% formalin for histological assays and the other was immediately frozen at -80°C for the MPO and cytokine assays.

### RNA Extraction and Real-Time Quantitative PCR

Total RNA was isolated from freshly biopsied colon tissues using the RNA simple Total RNA Kit (TIANGEN, Beijing, China) according to the manufacturer’s instructions. RT-qPCR was performed using 50 ng total RNA and a Quant One Step RT-qPCR Kit (SYBR Green) (TIANGEN, Beijing, China). The primer sequences used for qRT-PCR analyses are as follows: GAPDH (forward primer 5′-GAAGGTGAAGGTCGGAGTC-3′, reverse primer 5′-GAAGATGGTGATGGGATTTC-3′); XBP1 (forward primer 5′-GAGCAGCAAGTGGTGGATTT-3′, reverse primer 5′-AAAGGGAGGCTGGTAAGGAA-3′); NF-κB (forward primer 5′-TCTTCAACATGGCAGACGAC-3′, reverse primer 5′-CTCTCTGTTTCGGTTGCTCT). The reaction conditions were as follows: reverse transcription at 50°C for 30 min, followed by 40 cycles of pre-denaturation at 95°C for 2 min, denaturation at 94°C for 20 s, annealing at 58°C for 20 s, and extension at 68°C for 20 s. RNA expression was calculated as Ct, and relative expression levels were calculated as 2^-ΔΔC_*t*_^.

### Histopathological and Immunohistochemical (IHC) Evaluations

Colon tissues were fixed in 10% formalin, dehydrated using graded concentrations of ethanol, embedded in paraffin, and sectioned (5 μm thick). These sections were mounted on slides, cleared, and rehydrated. Tissue sections were stained with hematoxylin and eosin (HE) as per the standard method. Histological scores were obtained by grading the sections 0–3 based on epithelial injury and depth of ulceration, 0–3 based on edema, 0–3 based on infiltrating cells (lymphocytes, monocytes, and plasmocytes) and depth of infiltration, 0–3 for infiltration with neutrophils, and 0–3 for infiltration of eosinophils and infiltration depth (Kaser et al., 2008). For evaluation by IHC, IHC was performed according to standard protocols using anti-mouse XBP1 and anti-mouse NF-κB antibodies (Abcam, United States) at a 1:100 dilution.

### Biochemical Assays

Colon tissues stored at -80°C were weighed and processed as follows. All colon samples were immediately frozen in liquid nitrogen and then thawed. Then, the samples were homogenized in lysis buffer containing 50 mM Tris–HCl pH 8.0, 150 mM NaCl, 1 mM EDTA, 0.5% Triton X-100, and protease inhibitor (Roche) with a homogenizer. The colon homogenates were centrifuged at 12,000 × *g* at 4°C for 30 min, and the supernatants were collected. Supernatant protein concentrations were quantified using a BCA assay kit. MPO activity, TNF-α, IL-6, and IL-1β expression levels were measured according to the R&D kit instructions.

### Western-Blot

Cell lysates and supersonic lysates of colon tissues were processed for Western-blot as follows. First, the lysates were centrifuged at 12,000 *g* for 10 min and then quantified by BCA assay. Next, after boiling at 100°C for 5 min, the supernatants were separated and immunoblotted. The membrane was blocked in Tris-buffered saline with 0.1% Tween-20 (TBST) solution containing 5% non-fat dried milk for 1 h and then incubated overnight at 4°C with specific antibodies. After washing with TBST, the protein bands were labeled with anti-mouse IgG for 2 h at room temperature. Finally, the membranes were washed with TBST, and the protein bands were detected, photographed, and analyzed using Image J software.

### Statistical Analysis

Results were analyzed for statistical significance using one-way analysis of variance (ANOVA) followed by Tukey’s *post hoc* test. Data were expressed as mean ± SEM. *P*-values < 0.05 were considered statistically significant.

## Results

### (±)-8-ADC Significantly Increased Transcriptional Activation of XBP1 in IEC6 Cells

(±)-8-ADC was first investigated for potential cytotoxicity in IEC6 cells using the CCK8 assay (**Figure 1B**). It was found (±)-8-ADC displayed no cytotoxicity in IEC6 cells, where survival of (±)-8-ADC-treated IEC6 cells ranged from 97 to 101% compared to the untreated cells.

To examine the effect of (±)-8-ADC on transcription of XBP1, we constructed an XBP1 promoter reporter gene vector and introduced it into IEC6 cells. Following treatment of these cells with (±)-8-ADC for 24 h in 48-well plates, the luciferase activity of the transfected cells was measured. The effect of (±)-8-ADC on XBP1 activation is shown in **Figure 1C**, where C is the background contrast and C1 is the pGL3-basic vector control. Compared with C1 transfected cells, (±)-8-ADC (0.01, 0.1, and 1 μM) treated cells had increased activation of XBP1 in IEC6 cells in a dose-dependent manner.

### (±)-8-ADC Significantly Reduced Weight Loss in DSS-Induced Mice

First, acute toxicity of (±)-8-ADC in normal mice was assessed by orally administering different doses of (±)-8-ADC (up to 5 g/kg) for 2 weeks. The LD_50_ of (±)-8-ADC was determined to be about 4 g/kg. Next, the effect of treating with (±)-8-ADC for 7 days on DSS-induced UC was determined using double-blind observation, where the therapeutic effectiveness was evaluated based on several indicators as previously reported (Ben-Ami Shor et al., 2015; Raup-Konsavage et al., 2016).

**FIGURE 2:**
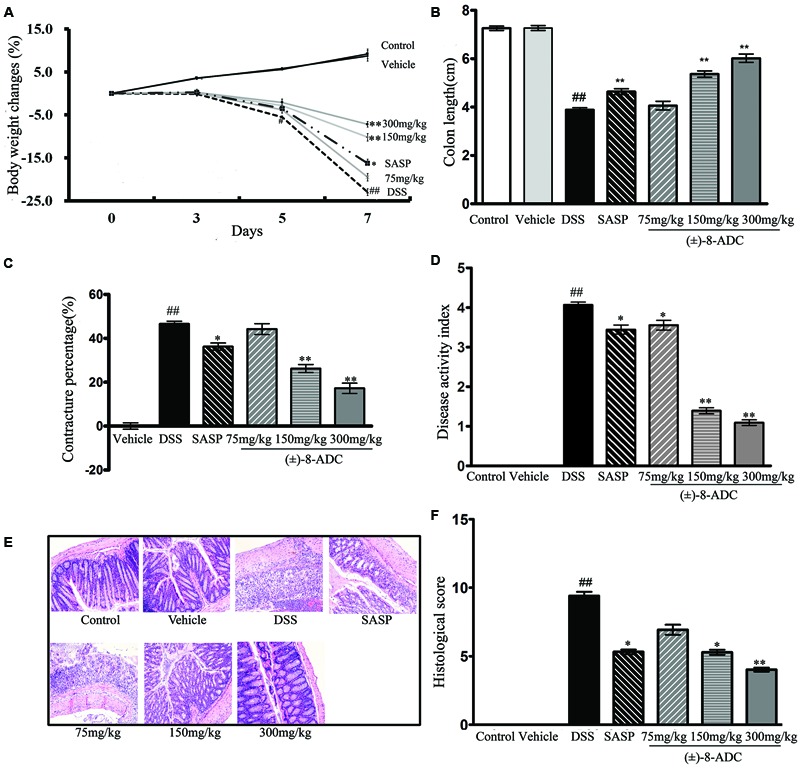
Effect of oral administration of (±)-8-ADC and SASP in DSS-induced mice (*n* = 6). **(A)** Treatment with 150 and 300 mg/kg (±)-8-ADC effectively prevented body weight loss in DSS-induced mice and had no effect in healthy mice. **(B)** Oral administration of (±)-8-ADC (150 and 300 mg/kg) for 7 days significantly ameliorated colon contracture in DSS-induced mice (*p* < 0.01). **(C)** Compared to the DSS group (contracture 46%), treatment with 150 and 300 mg/kg (±)-8-ADC reduced the colon contracture to 26 and 17%, respectively (*p* < 0.01). **(D)** Compared with the DSS group, all doses of orally administered (±)-8-ADC significantly decreased the DAI score. **(E)** Colon HE staining. Epithelial cell loss, submucosal ulceration, and a large number of infiltrating inflammatory cells were observed in the DSS-induced group. Treatment of these mice with (±)-8-ADC decreased this pathological damage in the colon tissue. **(F)** Colon histological scores. Treatment with (±)-8-ADC reduced the histological scores of the colons of DSS-induced mice compared to mock-treated DSS-induced mice. ^##^*p* < 0.01 compared with control group; ^∗^*p* < 0.05, ^∗∗^*p* < 0.01 compared with DSS group.

Oral administration of (±)-8-ADC ameliorated body weight loss in DSS-induced mice and had no discernable effect in normal mice (vehicle group). The DSS-induced mice lost approximately 20% of their initial body weight by 7 days post-DSS treatment in the DSS group. Treating DSS-induced mice with 150 or 300 mg/kg (±)-8-ADC significantly ameliorated weight loss in DSS-induced mice (**Figure 2A**). By contrast, there were no differences in mouse weight between the control and vehicle groups (*p* > 0.5) following treatment for 7 days (**Figure 2A**).

### (±)-8-ADC Alleviated Colon Contracture in DSS-Induced Mice

Consistent with the amelioration of weight loss, oral administration of (±)-8-ADC (150 and 300 mg/kg) significantly relieved colon contracture in DSS-induced mice (*p* < 0.01) after 7 days of treatment (**Figures 2B,C**). Compared to the DSS-induced group, where contracture averaged 46%, treatment with 150 and 300 mg/kg (±)-8-ADC reduced colon contracture to 26 and 17%, respectively. While SASP treatment decreased colon contracture, it was not as effective as (±)-8-ADC, as it only reduced it to 36%.

### (±)-8-ADC Decreased the DAI Scores of DSS-Induced UC Mice

The DAI was scored based on weight changes and the presence of loose and/or bloody stool. Seven days after colitis induction with DSS, an increased DAI score was observed mice (**Figure 2D**). However, all doses of (±)-8-ADC tested in DSS-induced mice significantly decreased the DAI scores (**Figure 2D**). Compared with the SASP group, the 150 and 300 mg/kg (±)-8-ADC treated groups had decreased DAI scores due to less colitis-induced weight loss, a normal stool consistency, and little to no visible blood in the stools.

### (±)-8-ADC Decreased Pathological Damage to Colon Tissue of DSS-Induced Mice

In line with evaluations of weight loss, colon length, and DAI, 150 and 300 mg/kg (±)-8-ADC also decreased pathological damage (**Figure 2E**), as well as the histological scores (**Figure 2F**). Inflammation and pathological changes were obvious throughout the mucosal epithelium and lamina propria of the colons of the DSS-induced mice, where edema, epithelial cell loss, submucosal ulceration, and a large number of infiltrating inflammatory cells, including neutrophils and mononuclear cells, were noted. Compared with the untreated DSS-induced group, 150 and 300 mg/kg (±)-8-ADC prevented inflammatory damage induced by DSS and reduced the histological scores of the colons to a greater extent than SASP treatment.

### (±)-8-ADC Increased XBP1 and Decreased NF-κB Expression Levels in the Colons of DSS-Induced Mice

**FIGURE 3:**
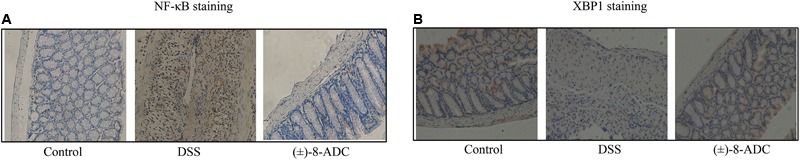
Analysis of NF-κB and XBP1 expression through IHC staining. **(A)** NF-κB expression in mouse colon tissue. Treatment with 300 mg/kg (±)-8-ADC decreased NF-κB expression compared with the mock-treated DSS-induced group. **(B)** XBP1 expression in mouse colon tissue. Treatment with 300 mg/kg (±)-8-ADC increased XBP1 expression compared with the mock-treated DSS-induced group.

**FIGURE 4:**
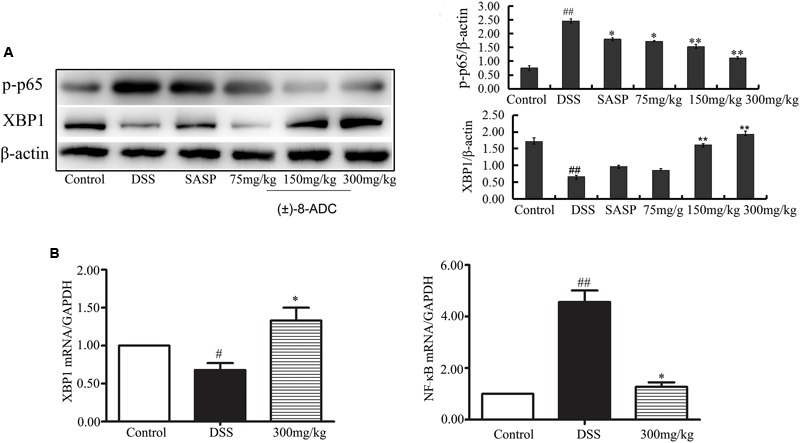
Effect of (±)-8-ADC treatment on NF-κB and XBP1 expression (*n* = 3). **(A)** Western-blot result and its quantitative analysis results. Treatment with (±)-8-ADC decreased NF-κB expression and increased XBP1 expression compared with the DSS-induced group in a dose-dependent manner. **(B)** Real-time quantitative PCR results. (±)-8-ADC decreased NF-κB mRNA expression and increased XBP1 mRNA expression compared with the DSS-induced group. ^#^*p* < 0.05, ^##^*p* < 0.01 compared with the control group; ^∗^*p* < 0.05, ^∗∗^*p* < 0.01 compared with the DSS-induced group.

Protein and mRNA expression changes in XBP1 and NF-κB were also observed in the colon tissues. IHC staining demonstrated that (±)-8-ADC treatment decreased NF-κB (**Figure 3A**) and increased XBP1 expression levels (**Figure 3B**) following DSS-induction of colitis. Western-blot revealed that (±)-8-ADC treatment also decreased p-p65 expression levels and increased XBP1 expression induced by DSS (**Figure 4A**). Furthermore, 150 and 300 mg/kg (±)-8-ADC reduced p-p65 expression levels to a greater extent than SASP treatment. At the transcription level, (±)-8-ADC decreased the NF-κB mRNA expression and increase the expression of XBP1 mRNA (**Figure 4B**).

### (±)-8-ADC Inhibited the Production of Pro-inflammatory Factors TNF-α, IL-1β, IL-6, and MPO

Myeloperoxidase levels are often used to quantify neutrophil functions and activity in inflamed tissues (Tran et al., 2007; Laroui et al., 2012). An increase in leukocyte adhesion to and accumulation in colon tissues is typically observed during UC. TNF-α, IL-1β and IL-6 are the important inflammatory cytokines and believed to promote the inflammatory response in patients with UC (Bressler et al., 2015).

**FIGURE 5:**
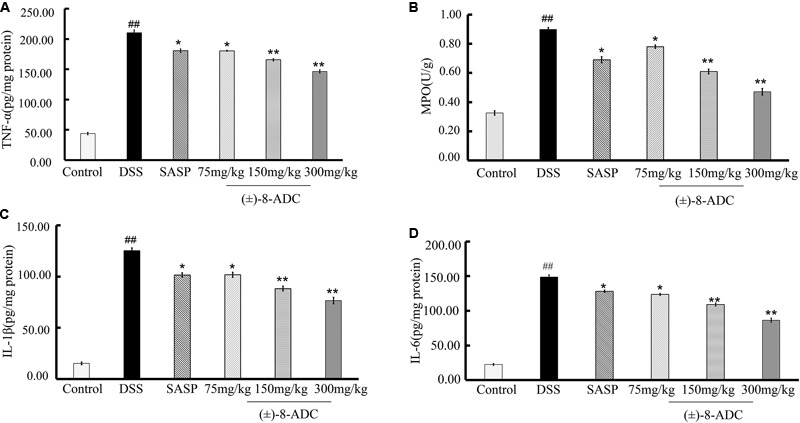
Effect of (±)-8-ADC on pro-inflammatory cytokine production in the colons of DSS-induced mice (*n* = 6). **(A)** TNF-α expression and **(B)** MPO activity in colon tissues. All doses of (±)-8-ADC tested reduced MPO activity in DSS-induced mouse colon tissue. **(C)** IL-1β and **(D)** IL-6 expression in colon tissues. All doses of (±)-8-ADC tested reduced TNF-α, IL-1β, and IL-6 expression in DSS-induced mouse colon tissues. ^##^*p* < 0.01 compared with the control group; ^∗^*p* < 0.05, ^∗∗^*p* < 0.01 compared with the DSS-induced group.

In our study, MPO activity, TNF-α, IL-1β, and IL-6 were measured. As previously reported, MPO activity, TNF-α, IL-1β, and IL-6 levels in colon tissues of DSS-induced mice were increased compared to non-DSS-induced mice (Tran et al., 2007; Laroui et al., 2012; Bressler et al., 2015). Orally administered (±)-8-ADC reduced TNF-α, IL-1β, IL-6, and MPO levels significantly (**Figure 5**). Compared with the DSS-induced group, TNF-α levels decreased by 14% (*P* < 0.05), 21% (*P* < 0.01), and 30% (*P* < 0.01) following treatment with 75, 150, and 300 mg/kg (±)-8-ADC, respectively (**Figure 5A**). IL-1β levels decreased by 19% (*P* < 0.05), 30% (*P* < 0.01), and 39% (*P* < 0.01) following treatment with 75, 150, and 300 mg/kg (±)-8-ADC, respectively (**Figure 5C**). IL-6 levels were reduced by 17% (*P* < 0.05), 27% (*P* < 0.01), and 42% (*P* < 0.01) by (±)-8-ADC treatment with 75, 150, and 300 mg/kg, respectively (**Figure 5D**). MPO levels were reduced by 13% (*P* < 0.05), 32% (*P* < 0.01), and 48% (*P* < 0.01) by treatment with 75, 150, and 300 mg/kg (±)-8-ADC, respectively (**Figure 5B**). In addition, (±)-8-ADC (150 and 300 mg/kg) was superior to treatment with SASP (300 mg/kg) in terms of TNF-α, IL-1β, IL-6, and MPO inhibition.

### (±)-8-ADC Influenced the XBP1-NF-κB Pathway in IEC6 Cells

**FIGURE 6:**
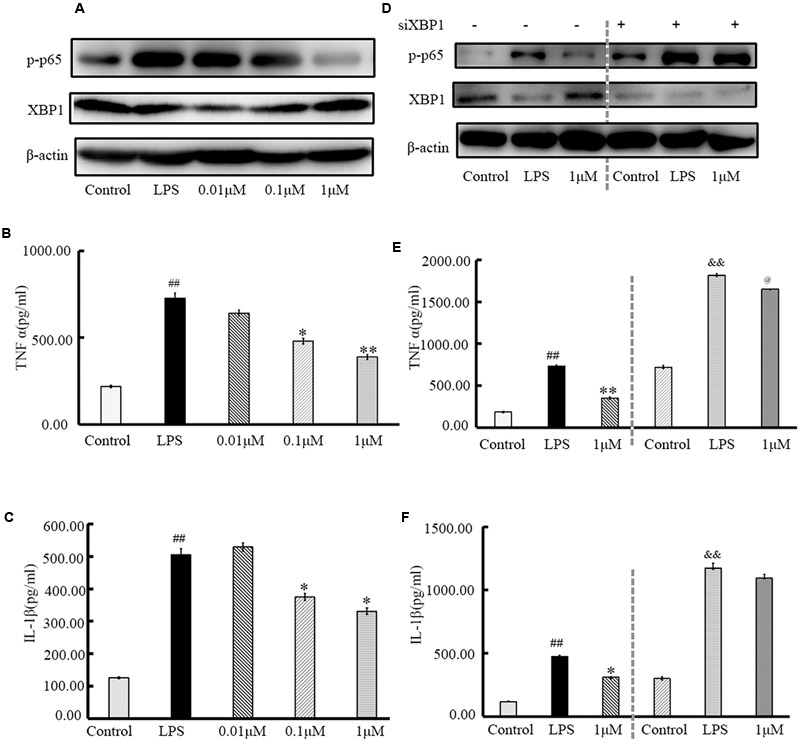
Treatment with (±)-8-ADC inhibits XBP1, NF-κB, and pro-inflammatory cytokine production in IEC6 cells. **(A)** Western-blot of IEC6 cells. The effect of (±)-8-ADC on NF-κB p-p65 phosphorylation was determined by Western-blot. (±)-8-ADC inhibited NF-κB p65 phosphorylation in IEC6 cells. **(B,C)** Effect of (±)-8-ADC on **(B)** TNF-α and **(C)** IL-1β production. Following stimulation with 5 μg/ml LPS for 24 h, (±)-8-ADC decreased TNF-α and IL-1β expression in IEC6 cells in a dose-dependent manner. **(D)** Western-blot of siXBP1 in IEC6 cells. In the negative control group, siXBP1(–), (±)-8-ADC inhibited p-p65 expression and increased XBP1 expression in IEC6 cells in a dose-dependent manner. Conversely, inhibition effect of (±)-8-ADC was reduced by silencing XBP1 in IEC6 cells. Compared with the negative control group, siXBP1(–), p-p65 was notably increased in siXBP1(+) groups. **(E,F)** The effect of (±)-8-ADC on **(E)** TNF-α and **(F)** IL-1β production following silencing of XBP1 in IEC6 cells. Compared with the negative control group, siXBP1(–), secretion of TNF-α and IL-1β were significantly higher in the siXBP1(+) groups. Each experiment was repeated three times. ^##^*p* < 0.01 compared with the control group; ^∗^*p* < 0.05, ^∗∗^*p* < 0.01 compared with the LPS group. ^&&^*p* < 0.01 compared with the “control+si” group, ^@^*p* < 0.05 compared with the “LPS+si” group.

Using qRT-PCR and Western blotting (**Figure 6**), p-p65 and XBP1 expression levels in IEC6 cells were assayed after treatment with (±)-8-ADC and LPS stimulation (5 μg/ml) for 24 h. Doses of 0.1 and 1 μM of (±)-8-ADC were found to effectively increase XBP1 and decrease p-p65 expression in a dose-dependent manner (**Figure 6A**). In addition, treatment with 0.1 and 1 μM (±)-8-ADC decreased TNF-α and IL-1β production (**Figures 6B,C**).

XBP1 gene silencing assays were carried out under the same conditions. After transfection with siXBP1 for 3 h, IEC6 cells were treated with 1 μM (±)-8-ADC and stimulated with LPS (5 μg/ml) for 24 h. Compared with the siXBP1(-) control groups, NF-κB expression (**Figure 6D**) and secretion of TNF-α and IL-1β (**Figures 6E,F**) were increased in the siXBP1(+) groups. Therefore, silencing XBP1 expression effectively blocked the effect of (±)-8-ADC on the NF-κB pathway and cytokine secretion.

## Discussion

In this study, we evaluated (±)-8-ADC activity against UC in a DSS-induced mouse model and made preliminary characterizations of its mechanisms of action. DSS-induced colitis is a well-established experimental model that simulates many of the features of human UC. As previously reported, the activity compounds against UC can be evaluated using several indicators, including DAI score, colon contracture, and pathological changes (Li et al., 2015; Tasaka et al., 2015). The DAI index is commonly used to describe changes in UC and takes into account weight loss, loose stools, and intestinal bleeding. In our study, oral administration of (±)-8-ADC had a significant effect on the DAI scores, as well as ameliorated colon contracture and histological damage related to colitis (**Figure 2**).

A number of cytokines regulate mucosal inflammation and affect function of the intestinal epithelium during UC pathogenesis. During the course of UC and experimental colitis, pro-inflammatory cytokines, such as TNF-α, are released that lead to exacerbation of tissue damage (Bravatà et al., 2015). Many studies have reported TNF-α is involved in UC progression. Sethuraman et al. (2015) demonstrated that a combination of emu oil and glycyrrhizin may act synergistically against UC by regulating TNF-α. Ślebioda and Kmieć (2014) showed in detail that TNF-α has a pathological role in inflammatory bowel disease. Higher levels of MPO can result in the production of cytotoxic reactive oxygen species, which can eventually lead to colon mucosal disruption and ulceration. In this present study, we measured changes in the levels of certain factors, including TNF-α, MPO, IL-1β, and IL-6, in response to (±)-8-ADC treatment. The levels of the pro-inflammatory cytokines TNF-α, IL-1β, and IL-6, and MPO in the DSS-induced group increased, which is in agreement with previous studies. Oral administration of (±)-8-ADC decreased the levels of TNF-α, IL-1β, IL-6 and MPO, which may be related to its activity against UC (**Figure 5**).

Salicylates, such as SASP, are the first-line treatments for UC in the clinic. However, these compounds have many side effects, such as gastrointestinal reactions, and require a comparably large dose (the standard dose for humans is 4000 mg/day) (Jiang and Cui, 2004; Feagan and Macdonald, 2012). Oral (±)-8-ADC has a comparable or even better effect than SASP in DSS-induced mice in terms of inhibiting UC. Compared to SASP, orally administered (±)-8-ADC (150 and 300 mg/kg) was more effective at treating DSS-induced UC mice, as shown by improvements in weight loss, colon contracture, pathological damage, DAI score, and levels of TNF-α, IL-1β, IL-6, and MPO in the colon tissues (**Figures 2, 4, 5**). At the same time, (±)-8-ADC had no toxic and side effect on normal mice including weight and so on in this study and displayed no long-term toxicity or cytotoxicity (**Figures 1B, 2**). Our results demonstrate that (±)-8-ADC may be a potential substitute for SASP in the clinic.

XBP1 and NF-κB play very important roles during the progression of inflammation, including in experimental colitis. Both inhibition of XBP1 activation and knockdown of XBP1 have been shown to aggravate inflammation, and overexpression of XBP1 results in a decrease in NF-κB activity and reduced cytokine expression (Wei and Feng, 2010; Li et al., 2011; Shaked et al., 2015; Yu et al., 2015; Zhu et al., 2017). Our results showed that (±)-8-ADC upregulated the transcriptional activation of XBP1 in IEC6 cells (**Figure 1**). In addition, (±)-8-ADC increased XBP1 expression, and reduced phospho-NF-κB p65 expression and proinflammatory secretion, which may consequently inhibit the inflammatory processes involved in UC (**Figures 3–5**). When XBP1 was silenced in IEC6 cells, expression of phospho-NF-κB p65 and proinflammatory secretion increased following with LPS stimulation compared with normal LPS stimulated IEC6 cells, revealing (±)-8-ADC inhibited the NF-κB pathway via XBP1 (**Figure 6**). We also observed the effect of (±)-8-ADC on NF-κB was not abrogated completely by silencing XBP1, and (±)-8-ADC had a suppressive effect on TNF-α secretion but no effect on IL-1β secretion. We speculate that (±)-8-ADC may be a multiple target compound and, thus, further studies on this are in progress. In addition, related research has shown that XBP1 links endoplasmic reticulum (ER) stress to intestinal inflammation (Kaser et al., 2008). ER stress and autophagy also take part in the pathogenesis of inflammatory bowel disease as interlinking pathways (Hosomi et al., 2015). Whether ER stress and autophagy have mainly roles in the inhibition of UC by (±)-8-ADC, and the mechanisms of action behind this have also attracted our great attention. Therefore, further experiments are now being carried out to elucidate the relationship between XBP1, ER stress, and autophagy.

Overall, (±)-8-ADC significantly inhibited the development of colitis and improved the pathology associated with acute colitis induced by DSS by acting via the XBP1-NF-κB pathway. In this study, orally administered (±)-8-ADC ameliorated weight loss and colon contracture, reduced DAI scores, prevented pathological damage, and decreased the expression of MPO and pro-inflammatory cytokines in DSS-induced mice and/or IEC6 cells. The effect of (±)-8-ADC on DSS-induced inflammation was related to its capacity to activate XBP1 transcriptional activity and inhibit NF-κB expression. Compared with currently available treatments, such as SASP (300 mg/kg), orally administered (±)-8-ADC (150 and 300 mg/kg) is more effective and safer. Therefore, the monomer (±)-8-ADC has potential for development as a therapeutic candidate to treat UC.

## Author Contributions

HZ and GS contributed to study design, and data collection and analysis. ZZ contributed to compound synthesis. HS and XT contributed to data collection. AD contributed to the literature search. LW, WW, and HQ contributed by creating the final version of the manuscript and through Funds Collection. All authors reviewed and approved the manuscript prior to submission.

## Conflict of Interest Statement

The authors declare that the research was conducted in the absence of any commercial or financial relationships that could be construed as a potential conflict of interest. The reviewer XT and handling Editor declared their shared affiliation, and the handling Editor states that the process met the standards of a fair and objective review.

## References

[B1] Adolph T. E. Tomczak M. F. Niederreiter L. Ko H. J. Böck J. Martinez-Naves E. (2013). Paneth cells as a site of origin for intestinal inflammation. *Nature* 503 272–276. 10.1038/nature12599 24089213 PMC3862182

[B2] Akiho H. Yokoyama A. Abe S. Nakazono Y. Murakami M. Otsuka Y. (2015). Promising biological therapies for ulcerative colitis: a review of the literature. *World J. Gastrointest. Pathophysiol.* 15 219–227. 10.4291/wjgp.v6.i4.219 PMC4644886 26600980

[B3] Bank S. Andersen P. S. Burisch J. Pedersen N. Roug S. Galsgaard J. (2014). Associations between functional polymorphisms in the NFκB signaling pathway and response to anti-TNF treatment in Danish patients with inflammatory bowel disease. *Pharmacogenomics J.* 14 26–34. 10.1038/tpj.2014.19 24776844

[B4] Ben-Ami Shor D. Bashi T. Lachnish J. Fridkin M. Bizzaro G. Barshak I. (2015). Phosphorylcholine-tuftsin compound prevents development of dextransulfate-sodium-salt induced murine colitis: implications for the treatment of human inflammatory bowel disease. *J. Autoimmun.* 56 111–117. 10.1016/j.jaut.2014.11.001 25479760

[B5] Bravatà I. Allocca M. Fiorino G. Danese S. (2015). Integrins and adhesion molecules as targets to treat inflammatory bowel disease. *Curr. Opin. Pharmacol.* 25 67–71. 10.1016/j.coph.2015.11.007 26687159

[B6] Bressler B. Marshall J. K. Bernstein C. N. Bitton A. Jones J. Leontiadis G. I. (2015). Clinical practice guidelines for the medical management of nonhospitalized ulcerative colitis: the Toronto consensus. *Gastroenterology* 148 1035–1058.e3. 10.1053/j.gastro.2015.03.001 25747596

[B7] Fan H. Liu X. X. Zhang L. J. Hu H. Tang Q. Duan X. Y. (2014). Intervention effects of QRZSLXF, a Chinese medicinal herb recipe, on the DOR-β-arrestin1-Bcl2 signal transduction pathway in a rat model of ulcerative colitis. *J. Ethnopharmacol.* 28 88–97. 10.1016/j.jep.2014.03.021 24637189

[B8] Feagan B. G. Macdonald J. K. (2012). Oral 5-aminosalicylic acid for induction of remission in ulcerative colitis. *Cochrane Database Syst. Rev.* 17:CD000543 10.1002/14651858.CD000543.pub3 23076889

[B9] Hosomi S. Kaser A. Blumberg R. S. (2015). Role of endoplasmic reticulum stress and autophagy as interlinking pathways in the pathogenesis of inflammatory bowel disease. *Curr. Opin. Gastroenterol.* 31 81–88. 10.1097/MOG.0000000000000144 25426970 PMC4592163

[B10] Jiang X. L. Cui H. F. (2004). Different therapy for different types of ulcerative colitis in China. *World J. Gastroenterol.* 15 1513–1520. 10.3748/wjg.v10.i10.1513 PMC4656295 15133864

[B11] Kaser A. Lee A. H. Franke A. Glickman J. N. Zeissig S. Tilg H. (2008). XBP1 links ER stress to intestinal inflammation and confers genetic risk for human inflammatory bowel disease. *Cell* 134 743–756. 10.1016/j.cell.2008.07.021 18775308 PMC2586148

[B12] Laroui H. Ingersoll S. A. Liu H. C. Baker M. T. Ayyadurai S. Charania M. A. (2012). Dextran sodium sulfate (DSS) induces colitis in mice by forming nano-lipocomplexes with medium-chain-length fatty acids in the colon. *PLoS ONE* 7:e32084 10.1371/journal.pone.0032084 PMC3302894 22427817

[B13] Lee J. W. Iwahashi A. Hasegawa S. Yonezawa T. Jeon W. B. Cha B. Y. (2012). Coptisine inhibits RANKL-induced NF-κB phosphorylation in osteoclast precursors and suppresses function through the regulation of RANKL and OPG gene expression in osteoblastic cells. *J. Nat. Med.* 66 8–16. 10.1007/s11418-011-0537-7 21656335

[B14] Li J. Wang J. J. Zhang S. X. (2011). Preconditioning with endoplasmic reticulum stress mitigates retinal endothelial inflammation via activation of X-box binding protein 1. *J. Biol. Chem.* 286 4912–4921. 10.1074/jbc.M110.199729 21138840 PMC3039327

[B15] Li Y. H. Zhang M. Xiao H. T. Fu H. B. Ho A. Lin C. Y. (2015). Addition of berberine to 5-aminosalicylic acid for treatment of dextran sulfate sodium-induced chronic colitis in C57BL/6 mice. *PLoS ONE* 7:e0144101 10.1371/journal.pone.0144101 PMC4671595 26642326

[B16] Luo Y. Zhao H. Liu Z. Ju D. He X. Xiao C. (2010). Comparison of the enteric mucosal immunomodulatory activity of combinations of *Coptis chinensis* Franch. Rhizomes and *Evodia rutaecarpa* (Juss.) Benth. Fruits in mice with dextran sulphate sodium-induced ulcerative colitis. *Planta Med.* 76 766–772. 10.1055/s-0029-1240701 20033867

[B17] Novak G. Hindryckx P. Khanna R. Jairath V. Feagan B. G. (2017). The safety of vedolizumab for the treatment of ulcerative colitis. *Expert Opin. Drug Saf.* 16 501–507. 10.1080/14740338.2017.1300251 28276855

[B18] Pedersen J. Coskun M. Soendergaard C. Salem M. Nielsen O. H. (2014). Inflammatory pathways of importance for management of inflammatory bowel disease. *World J. Gastroenterol.* 7 64–77. 10.3748/wjg.v20.i1.64 PMC3886034 24415859

[B19] Peppercorn M. A. (1984). Sulfasalazine: pharmacology, clinical use, toxicity, and related new drug development. *Ann. Intern. Med.* 101 377–386. 10.7326/0003-4819-101-3-377 6147110

[B20] Raup-Konsavage W. M. Cooper T. K. Yochum G. S. (2016). A role for MYC in lithium-stimulated repair of the colonic epithelium after DSS-induced damage in mice. *Dig. Dis. Sci.* 61 410–422. 10.1007/s10620-015-3852-0 26320084

[B21] Ren T. Tian T. Feng X. Ye S. Wang H. Wu W. (2015). An adenosine A3 receptor agonist inhibits DSS-induced colitis in mice through modulation of the NF-κB signaling pathway. *Sci. Rep.* 12 9047 10.1038/srep09047 PMC4357005 25762375

[B22] Rogler G. (2015). Where are we heading to in pharmacological IBD therapy? *Pharmacol. Res.* 100 220–227. 10.1016/j.phrs.2015.07.005 26277232

[B23] Sands B. E. (2014). New drugs on the horizon for IBD. *Dig. Dis.* 32 74–81. 10.1159/000367832 25531356

[B24] Sethuraman S. N. Swaminathan S. Nelson S. B. Palaninathan P. S. Gopalan T. K. Velayudham P. (2015). Modulation of PPARγ and TNFα by emu oil and glycyrrhizin in ulcerative colitis. *Inflammopharmacology* 23 47–56. 10.1007/s10787-014-0226-8 25560991

[B25] Shaked H. Guma M. Karin M. (2015). Analysis of NF-κB activation in mouse intestinal epithelial cells. *Methods Mol. Biol.* 1280 593–606. 10.1007/978-1-4939-2422-6_35 25736774

[B26] Ślebioda T. J. Kmieć Z. (2014). Tumour necrosis factor superfamily members in the pathogenesis of inflammatory bowel disease. *Mediators Inflamm.* 2014:325129 10.1155/2014/325129 PMC4087264 25045210

[B27] Tasaka Y. Yasunaga D. Kiyoi T. Tanaka M. Tanaka A. Suemaru K. (2015). Involvement of stimulation of α7 nicotinic acetylcholine receptors in the suppressive effect of tropisetron on dextran sulfate sodium-induced colitis in mice. *J. Pharmacol. Sci.* 127 275–283. 10.1016/j.jphs.2014.12.016 25837923

[B28] Tran C. D. Ball J. M. Sundar S. Coyle P. Howarth G. S. (2007). The role of zinc and metallothionein in the dextran sulfate sodium-induced colitis mouse model. *Dig. Dis. Sci.* 52 2113–2121. 10.1007/s10620-007-9765-9 17410436

[B29] Wei J. Feng J. (2010). Signaling pathways associated with inflammatory bowel disease. *Recent Pat. Inflamm. Allergy Drug Discov.* 4 105–117. 10.2174/187221310791163071 20001899

[B30] Yu Y. Zhang L. Liu Q. Tang L. Sun H. Guo H. (2015). Endoplasmic reticulum stress preconditioning antagonizes low-density lipoprotein-induced inflammation in human mesangial cells through upregulation of XBP1 and suppression of the IRE1α/IKK/NF-κB pathway. *Mol. Med. Rep.* 11 2048–2054. 10.3892/mmr.2014.2960 25405329

[B31] Zhang Z. H. Zhang H. J. Deng A. J. Wang B. Li Z. H. Liu Y. (2015). Synthesis and structure-activity relationships of quaternary coptisine derivatives as potential anti-ulcerative colitis agents. *J. Med. Chem.* 24 7557–7571. 10.1021/acs.jmedchem.5b00964 26321079

[B32] Zhu S. Liu H. Sha H. Qi L. Gao D. S. Zhang W. (2017). PERK and XBP1 differentially regulate CXCL10 and CCL2 production. *Exp. Eye Res.* 155 1–14. 10.1016/j.exer.2017.01.002 28065589 PMC5359061

[B33] Zou Z. Y. Hu Y. R. Ma H. Wang Y. Z. He K. Xia S. (2015). Coptisine attenuates obesity-related inflammation through LPS/TLR-4-mediated signaling pathway in Syrian golden hamsters. *Fitoterapia* 105 139–146. 10.1016/j.fitote.2015.06.005 26073947

