# Supplementary material for: Colitis Is Effectively Ameliorated by (±)-8-Acetonyl-dihydrocoptisine via the XBP1-NF-κB Pathway
Source: Front Pharmacol. 2017 Sep 5;8:619. doi: 10.3389/fphar.2017.00619 (PMC5591823; doi:10.3389/fphar.2017.00619)
Supplement: Supplementary file 1 [file Data_Sheet_1.PDF]

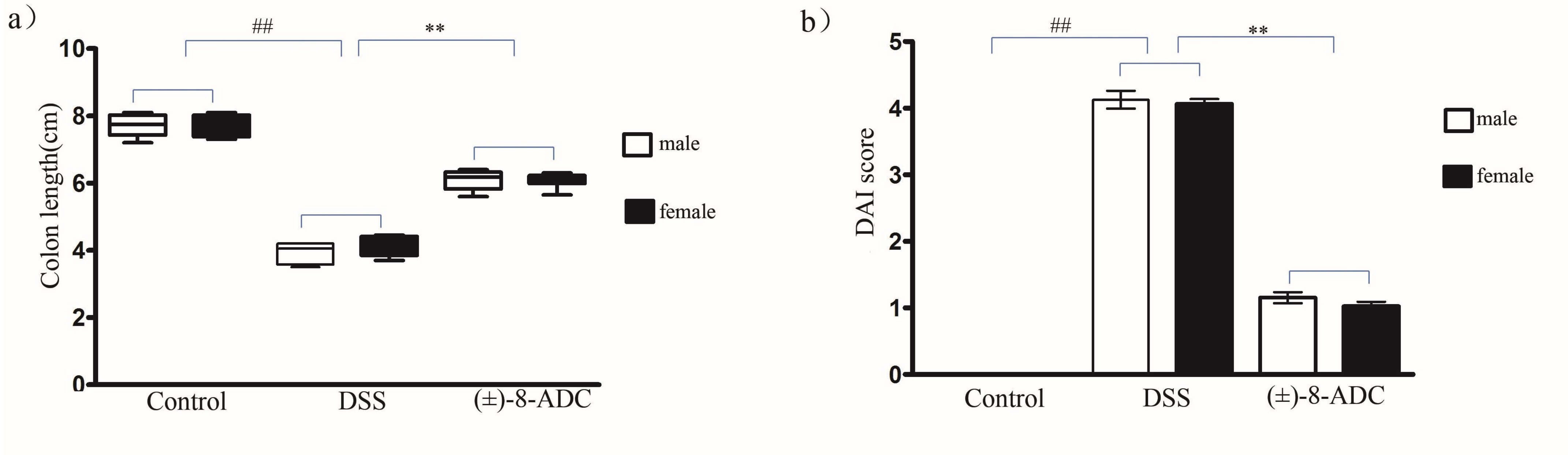

Supporting figure 1. Effect of oral administration of (±)-8-ADC on male and female mice (n=5). (a) Treatment with 300 mg/kg (±)-8-ADC effectively prevented the colon contracture in DSS-induced mice ($p < 0.01$). The effect of (±)-8-ADC on colon length had no difference in male and female mice. (b) Oral administration of (±)-8-ADC (300 mg/kg) for 7 days significantly decreased the DAI score in DSS-induced mice ($p < 0.01$). The effect of (±)-8-ADC had no difference in male and female mice. ##$p < 0.01$ compared with control group; **$p < 0.01$ compared with DSS group.

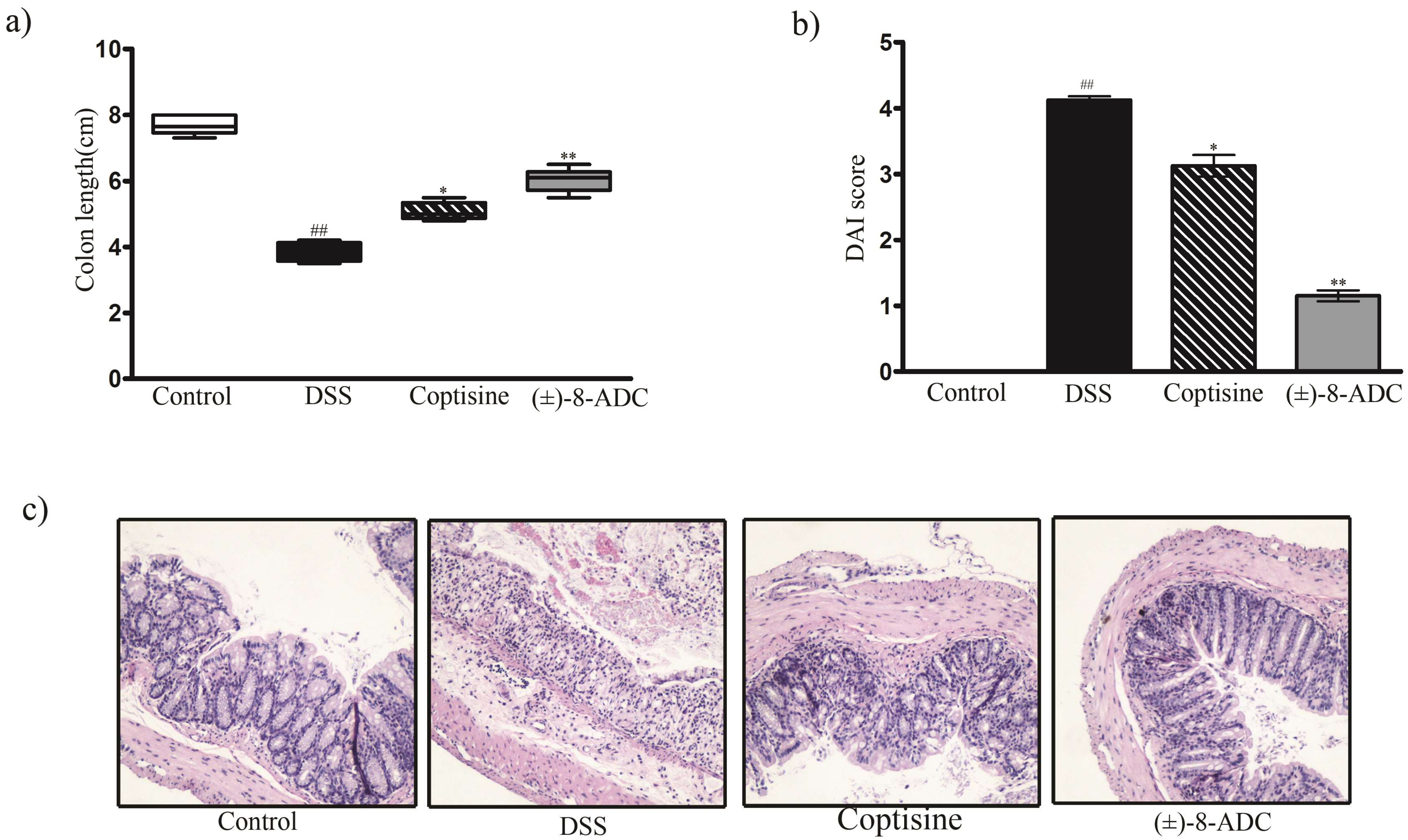

Supporting figure 2. Effect of (±)-8-ADC (300 mg/kg) and coptisine (300 mg/kg) against UC in mice. (a) (±)-8-ADC reduced the colon contracture better than coptisine (300 mg/kg) in DSS-induced mice. (b) (±)-8-ADC for 7 days significantly decreased the DAI score much better than coptisine in DSS-induced mice. (c) Colon HE staining. Compared with DSS group, (±)-8-ADC improved pathological damage much better than coptisine in the colon tissue. ##$p < 0.01$ compared with control group; *$p < 0.05$, **$p < 0.01$ compared with DSS group.
