# Supplementary material for: Colitis Is Effectively Ameliorated by (±)-8-Acetonyl-dihydrocoptisine via the XBP1-NF-κB Pathway
Source: Front Pharmacol. 2017 Sep 5;8:619. doi: 10.3389/fphar.2017.00619 (PMC5591823; doi:10.3389/fphar.2017.00619)
Supplement: Supplementary file 2 [file Data_Sheet_2.PDF]

中华人民共和国国家知识产权局

100088

北京市海淀区学院路蓟门里和景园A座1单元102室
北京三高永信知识产权代理有限责任公司 何文彬

发文日：

2013年05月30日

申请号或专利号：201310207277.7　　　　发文序号：2013053000512370

专利申请受理通知书

根据专利法第28条及其实施细则第38条、第39条的规定，申请人提出的专利申请已由国家知识产权局受理。现将确定的申请号、申请日、申请人和发明创造名称通知如下：

申请号：201310207277.7

申请日：2013年05月29日

申请人：中国医学科学院药物研究所

发明创造名称：黄连碱类生物碱衍生物及其抗溃疡性结肠炎的用途

经核实，国家知识产权局确认收到文件如下：

发明专利请求书 每份页数:5页 文件份数:1份

权利要求书 每份页数:4页 文件份数:1份 权利要求项数： 8项

说明书 每份页数:28页 文件份数:1份

说明书附图 每份页数:1页 文件份数:1份

说明书摘要 每份页数:1页 文件份数:1份

专利代理委托书 每份页数:2页 文件份数:1份

提示：

1. 申请人收到专利申请受理通知书之后，认为其记载的内容与申请人所提交的相应内容不一致时，可以向国家知识产权局请求更正。

2. 申请人收到专利申请受理通知书之后，再向国家知识产权局办理各种手续时，均应当准确、清晰地写明申请号。

审 查 员：黄瑞宏(电子申请)　　　　审查部门：专利局初审及流程管理部-15

200101 纸件申请，回函请寄：100088 北京市海淀区蓟门桥西土城路6号 国家知识产权局受理处收
2010.2 电子申请，应当通过电子专利申请系统以电子文件形式提交相关文件。除另有规定外，以纸件等其他形式提交的文件视为未提交。

发 明 专 利 请 求 书

代理机构内部编号 13SG1F0805

此框内容由国家知识产权局填写
①申请号 （发明）
②分案提交日
③申请日
④费减审批
⑤向外申请审批
⑥挂号号码

⑦发明名称	黄连碱类生物碱衍生物及其抗溃疡性结肠炎的用途		
⑧发明人	发明人 1	秦海林	☐不公布姓名
	发明人 2	王文杰	☐不公布姓名
	发明人 3	张志辉	☐不公布姓名
⑨第一发明人国籍	中国	居民身份证件号码	

⑩申请人	申请人(1)	姓名或名称：中国医学科学院药物研究所	用户代码	申请人类型 科研单位
		居民身份证件号码或组织机构代码 40000574-0	电子邮箱	
		国籍或注册国家（地区） 中国		
		省、自治区、直辖市 北京市		
		市县 宣武区		
		城区（乡）、街道、门牌号南纬路甲 2号		
		经常居所地或营业所所在地 中国	邮政编码 100050	电话
	申请人(2)	姓名或名称：	用户代码	申请人类型
		居民身份证件号码或组织机构代码		
		国籍或注册国家（地区）		
		省、自治区、直辖市		
		市县		
		城区（乡）、街道、门牌号		
		经常居所地或营业所所在地	邮政编码	电话
	申请人(3)	姓名或名称：	用户代码	申请人类型
		居民身份证件号码或组织机构代码		
		国籍或注册国家（地区）		
		省、自治区、直辖市		
		市县		
		城区（乡）、街道、门牌号		
		经常居所地或营业所所在地	邮政编码	电话

发 明 专 利 请 求 书

<table>
<tr><td rowspan="5">⑪联系人</td><td>姓　　名</td><td>电　　话</td></tr>
<tr><td>邮政编码</td><td>电子邮箱</td></tr>
<tr><td colspan="2">省、自治区、直辖市</td></tr>
<tr><td colspan="2">市县</td></tr>
<tr><td colspan="2">城区（乡）、街道、门牌号</td></tr>
</table>

⑫代表人为非第一署名申请人时声明	特声明第　　署名申请人为代表人

<table>
<tr><td rowspan="4">⑬专利代理机构</td><td colspan="2">名称北京三高永信知识产权代理有限责任公司</td><td colspan="2">机构代码 11138</td></tr>
<tr><td rowspan="3">代理人(1)</td><td>姓　　名何文彬</td><td rowspan="3">代理人(2)</td><td>姓　　名</td></tr>
<tr><td>执业证号 1113801354.9</td><td>执业证号</td></tr>
<tr><td>电　　话 010-62042352</td><td>电　　话</td></tr>
</table>

⑭分案申请	原申请号	针对的分案申请号	原申请日　　年　　月　　日

<table>
<tr><td rowspan="2">⑮生物材料样品</td><td>保藏单位</td><td colspan="2">地址</td></tr>
<tr><td>保藏日期　　年　　月　　日</td><td>保藏编号</td><td>分类命名</td></tr>
</table>

⑯序列表	☐ 本专利申请涉及核苷酸或氨基酸序列表
⑰遗传资源	☐本专利申请涉及的发明创造是依赖于遗传资源完成的

<table>
<tr><td rowspan="9">⑱要求优先权声明</td><td>序号</td><td>原受理机构名称</td><td>在先申请日</td><td>在先申请号</td><td rowspan="9">⑲不丧失新颖性宽限期声明</td><td rowspan="9">☐已在中国政府主办或承认的国际展览会上首次展出
☐已在规定的学术会议或技术会议上首次发表
☐他人未经申请人同意而泄露其内容</td></tr>
<tr><td>1</td><td></td><td></td><td></td></tr>
<tr><td>2</td><td></td><td></td><td></td></tr>
<tr><td>3</td><td></td><td></td><td></td></tr>
<tr><td>4</td><td></td><td></td><td></td></tr>
<tr><td>5</td><td></td><td></td><td></td></tr>
<tr><td>6</td><td></td><td></td><td></td></tr>
<tr><td>7</td><td></td><td></td><td></td></tr>
<tr><td>8</td><td></td><td></td><td></td></tr>
</table>

⑳保密请求	根据国家相关法律，涉及国家秘密的信息不得在国际联网的计算机信息系统中存储、处理、传递，故任何单位和个人认为其专利申请需要按照保密专利申请处理的，不得通过电子专利申请系统提交。
㉑同日申请	☐声明本申请人对同样的发明创造在申请本发明专利的同日申请了实用新型专利
㉒提前公布	☐请求早日公布该专利申请

发 明 专 利 请 求 书

㉓申请文件清单<br>1.发明专利请求书 共5页<br>2.说明书摘要 共0页<br>3.权利要求书 共0页<br>4.说明书 共0页<br>5.说明书附图 共0页<br><br>权利要求的项数 8 项	㉔附加文件清单<br>1.专利代理委托书 共2页<br><br>总委托书 (编号 014992)<br>证明文件备案编号
㉕全体申请人或专利代理机构签章<br><br>北京三高永信知识产权代理有限责任公司<br><br>2013年5月28日	㉖国家知识产权局审核意见<br><br>年 月 日

发 明 专 利 请 求 书 英 文 信 息 表

<table>
<tr><td>发明名称</td><td colspan="2"></td></tr>
<tr><td rowspan="3">发明人姓名</td><td>发明人 1</td><td></td></tr>
<tr><td>发明人 2</td><td></td></tr>
<tr><td>发明人 3</td><td></td></tr>
<tr><td rowspan="3">申请人名称及地址</td><td>申请人 1</td><td>名称
地址</td></tr>
<tr><td>申请人 2</td><td>名称
地址</td></tr>
<tr><td>申请人 3</td><td>名称
地址</td></tr>
</table>

附页

【发明人】

发明人 4	吴练秋	☐不公布姓名
发明人 5	邓安珺	☐不公布姓名
发明人 6	于金倩	☐不公布姓名
发明人 7	李志宏	☐不公布姓名

【发明人英文信息】

发明人 4	
发明人 5	
发明人 6	
发明人 7	

说 明 书 摘 要

本发明涉及以黄连碱季铵盐为原料经衍生化反应获得的新的 13-取代黄连碱季铵盐类衍生物、13-取代二氢黄连碱类生物碱衍生物和 13-取代四氢黄连碱类生物碱衍生物、其制备方法及其药物用途；其药物用途特征是：所述黄连碱类生物碱衍生物或其生理上可接受的盐在分子水平的活性测试实验中分别显示出一定的或较显著的 *xbp*1 启动子转录激活效应，是在治疗溃疡性结肠炎方面具有较高药用价值的化合物。

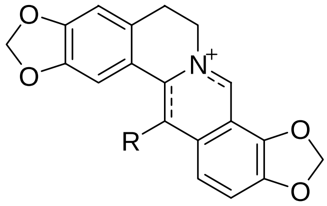

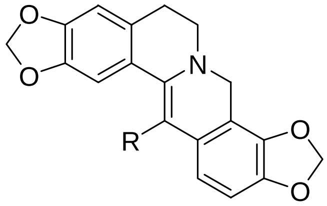

1、如通式Ⅰ所示的黄连碱类生物碱季胺盐衍生物：

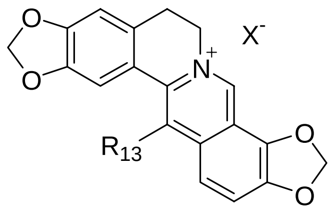

Ⅰ

其中，X^-为季铵盐负离子，可选自卤素负离子、硫酸根负离子；

R_{13}选自CH_2OR_{13}'并且R_{13}'选自H或脂肪烃基，并且R_{13}'烃基通式为C_nH_{2n+1}，n选自1-13的整数，或R_{13}'脂肪烃基通式为C_mH_{2m-1}，m选自2-13的整数；

或R_{13}选自$COOR_{13}$'并且R_{13}'选自H或烃基，并且R_{13}'烃基通式为C_nH_{2n+1}，n选自1-5的整数；

或R_{13}选自CH_2COOR_{13}'并且R_{13}'选自H或烃基，并且R_{13}'烃基通式为C_nH_{2n+1}，n选自1-5的整数。

2、如通式Ⅱ所示的黄连碱类生物碱季胺盐衍生物：

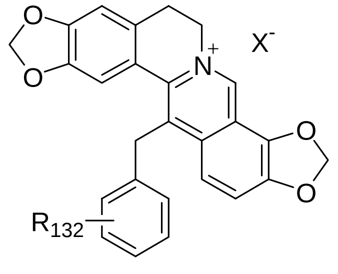

Ⅱ

其中，X^-为季铵盐负离子，可选自卤素负离子、硫酸根负离子等；R_{132}选自H、三氟甲基、氰基、羟基、氨基、硝基、苯基、亚甲二氧基、1,2-亚乙二氧基、卤素、C1-C4的烷基，C1-C4的烷氧基，C1-C4的烷硫基，C1-C4的烷酰基、C1-C4的烷酰氧基；上述C1-C4的烷基，C1-C4的烷氧基，C1-C4的烷硫基，C1-C4的烷酰基、C1-C4的烷酰氧基中的烷基是直链或支链。

3、如通式III所示的二氢黄连碱类生物碱衍生物：

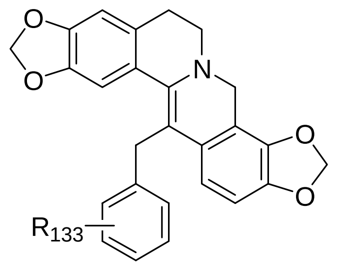

III

其中，R_{133}选自H、三氟甲基、氰基、羟基、氨基、硝基、苯基、亚甲二氧基、1,2-亚乙二氧基、卤素、C1-C4的烷基，C1-C4的烷氧基，C1-C4的烷硫基，C1-C4的烷酰基、C1-C4的烷酰氧基；上述C1-C4的烷基，C1-C4的烷氧基，C1-C4的烷硫基，C1-C4的烷酰基、C1-C4的烷酰氧基中的烷基是直链或支链。

4、如通式IV所示的四氢黄连碱类生物碱衍生物：

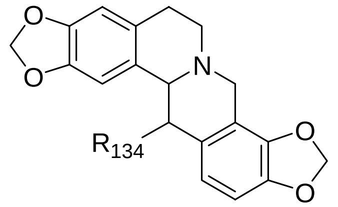

IV

其中， R_{134}选自CH_2COR_{13}'并且R_{13}'选自OH或烃氧基，并且R_{13}'烃氧基通式为OC_nH_{2n+1}，n选自1-13的整数。

5、如通式V所示的四氢黄连碱类生物碱衍生物：

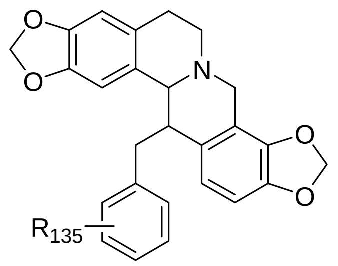

V

其中，R_{135}选自H、三氟甲基、氰基、羟基、氨基、硝基、苯基、亚甲二氧基、1,2-亚乙二氧基、卤素、C1-C4的烷基，C1-C4的烷氧基，C1-C4的烷硫基，C1-C4的烷酰基、C1-C4的烷酰氧基；上述C1-C4

的烷基，C1-C4 的烷氧基，C1-C4 的烷硫基，C1-C4 的烷酰基、C1-C4 的烷酰氧基中的烷基是直链或支链。

6、如权利要求 1-5 所述的各种黄连碱类生物碱衍生物或其生理上可接受的盐，其特征在于所述化合物选自如下式 **1-24** 新化合物群组中之一所示的结构：

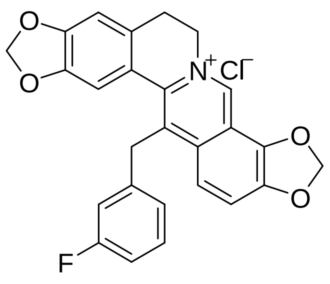

1

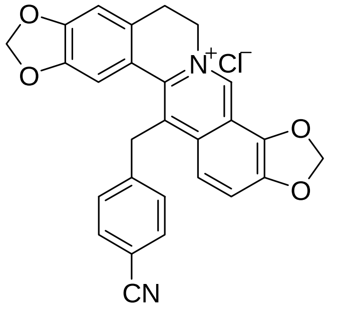

2

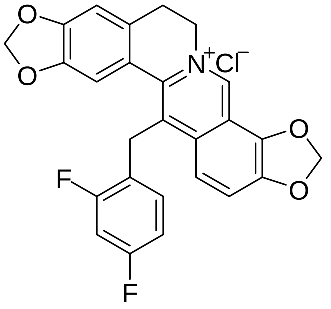

3

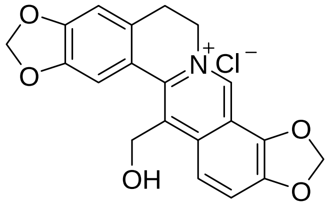

4

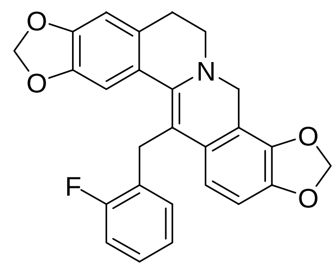

5

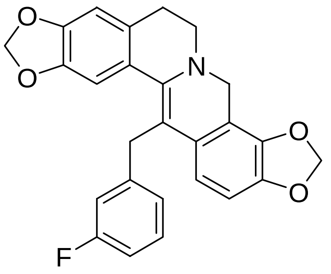

6

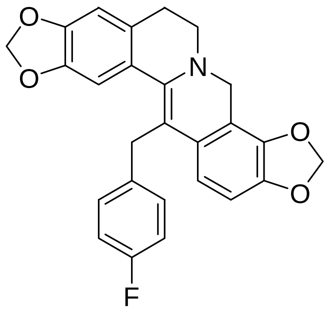

7

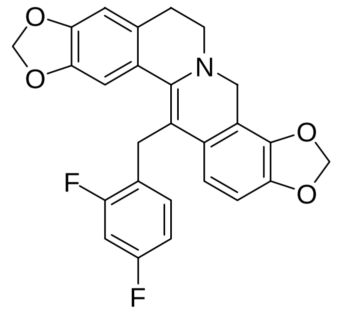

8

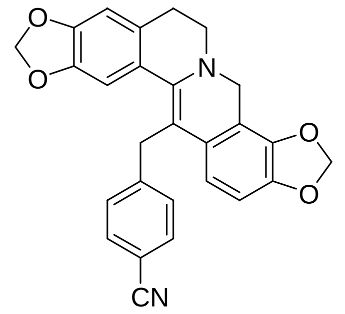

9

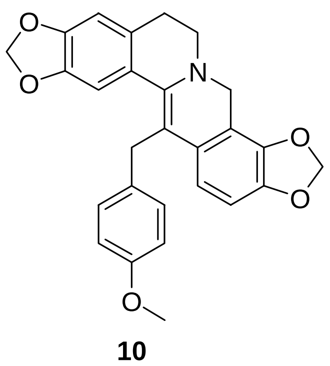

10

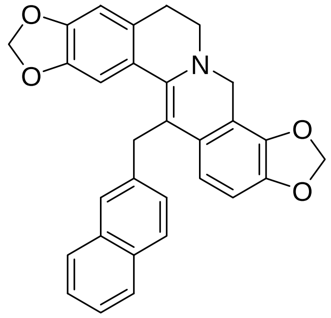

11

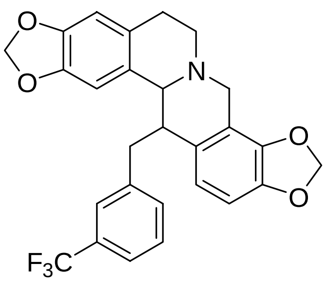

12

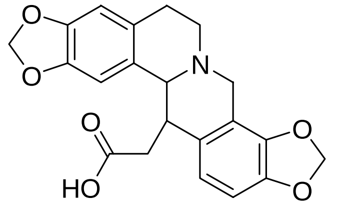

13

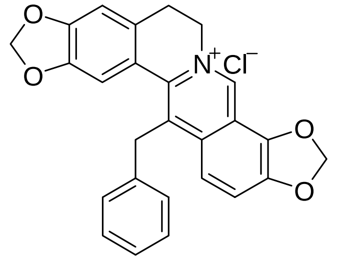

14

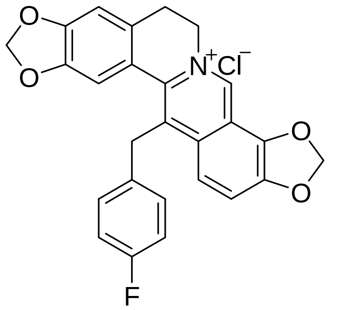

15

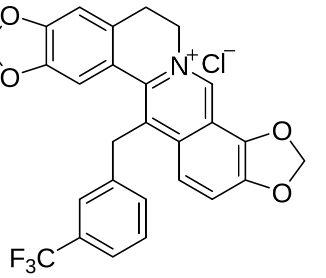

16

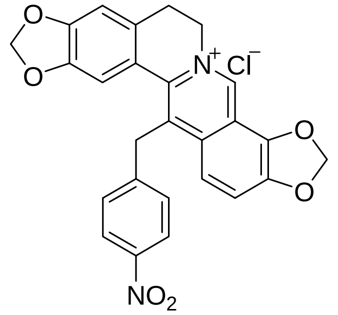

17

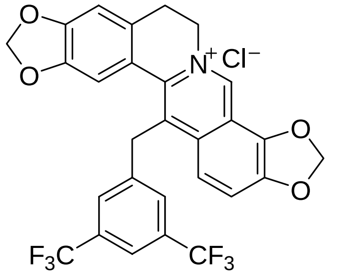

18

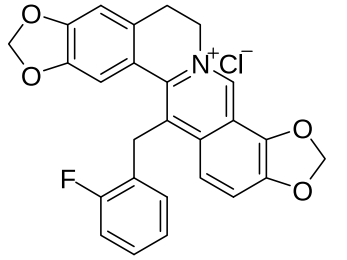

19

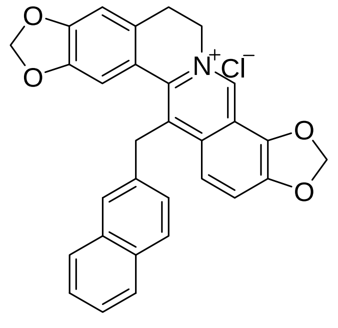

20

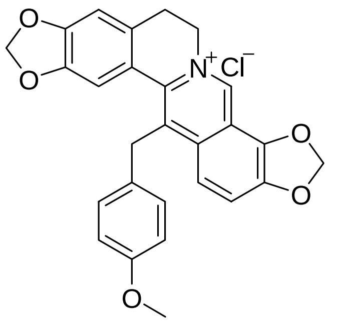

21

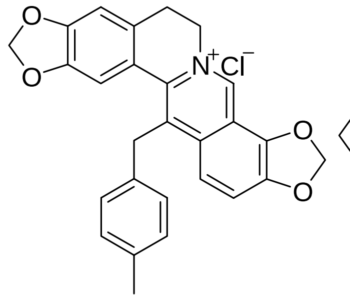

22

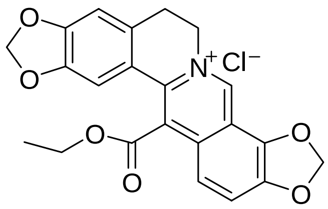

23

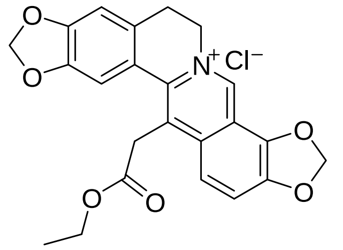

24

7、一种药物组合物，其特征在于，含有有效剂量的权利要求 1-6 中任一项的化合物和常用的药用载体。

8、权利要求 1-6 中任一项的化合物在制备治疗溃疡性结肠炎药物中的应用。

说　　明　　书

黄连碱类生物碱衍生物及其抗溃疡性结肠炎的用途

技术领域

本发明涉及以黄连碱季铵盐为底物经多种衍生化反应获得的新的黄连碱类生物碱衍生物、其制备方法及其抗溃疡性结肠炎的药物用途。具体黄连碱类生物碱衍生物或其生理上可接受的盐包括 13-取代黄连碱季铵盐类衍生物、13-取代二氢黄连碱类生物碱衍生物和 13-取代四氢黄连碱类生物碱衍生物。本发明属属于医药技术领域。

背景技术

溃疡性结肠炎(Ulcerative colitis，UC)属慢性非特异性炎性肠道疾病，临床上具有迁延发作、难于根治、亦伴发恶变等特点，是一种公认的严重影响易患人群生活质量甚至威胁生存的恶性疾病。我国 UC 临床上发病率为每年 1.2-20.2 例 / 10 万人，并且其患病率每年 7.6-206 例 / 10 万人；从世界范围看，北欧和北美的人群发病率更高，而亚洲地区发病率相对较低；但我国溃疡性结肠炎发病率有逐年上升趋势，其原因有待探讨[池肇春，溃疡性结肠炎发病的分子机制进展，中国医师进修杂志, 2012, 35（19）1-3]。此外，部分 UC 病人会出现恶性变，最终发展为 UC 癌性变，出现肠道腺体重度发育不良和结、直肠的肿瘤。目前在对 UC 的治疗中，即便是在达到显效的治疗方面，也极其缺少药物和其它疗法，更没有根治的方法；临床仅有美沙拉嗪（5-氨基水杨酸，为 SASP 治疗溃疡性结肠炎的活性成分）、免疫抑制剂和类固醇激素等少数几类药物。它们虽具有一定疗效，但仍经常复发，并各自存在较严重的不良反应。因此，寻找和发现特异性高、治疗效果佳

和副作用小的抗 UC 新药具有显著的必要性。

在医学上，UC 的病因和发病机制虽有多种学说，但至今仍未完全明确。本发明从 UC 属于炎性肠道疾病(Inflammatory bowel disease, IBD)的范畴出发，通过广泛查阅文献，探讨 UC 的病因，为建立新的药理模型奠定基础。目前临床上较常见的 IBD 包括克罗恩病(Crohn’s disease，CD)和溃疡性结肠炎, 都具有发病率高、病程长、病情反复发作等临床特点。虽然经过几十年的研究与观察，学术界有观点认为此两种疾病在病因学、免疫学、分子机制、病理及治疗等方面有许多差异，但也存在许多共同点。一项新近的研究发现，基因因素可能是诱发 IBD 的重要病因：与肠道上皮细胞内的非可控性内质网应激反应相关的下游关键转录因子 X-box-binding protein 1 (缩写为 *xbp*1)的功能异常与 IBD 的发病有着密切联系(Kaser A et al, Cell 2008, 134(5):743-756)。患有 IBD 的病人通常在 *xbp*1 基因的编码区会出现某些变异，从而使病人对 IBD 的诱发因素愈发敏感。*xbp*1 表达的缺失或下调会促进 IBD 的发生和加重 IBD 病情的发展。因此，*xbp*1 可能成为治疗 IBD 潜在的、新的药物作用靶点。然而，目前有关治疗 IBD 药物研发中以 *xbp*1 作为药物作用靶点的化学药物单体还未见报道。

本发明通过建立以 *xbp*1 基因为作用靶点的体外药物筛选模型，结合双荧光素酶报告基因、实时定量聚合酶链式反应（Polymerase Chain Reaction，PCR）和蛋白质印迹（Western Blot）法等细胞和分子生物学研究手段，从 *xbp*1 的基因转录调控、mRNA 表达及蛋白合成等不同层面寻找并发现 *xbp*1 的选择性激动剂。

本发明通过对黄连碱季铵盐进行结构修饰，得到了一系列黄连碱类生物碱衍生物或其生理上可接受的盐，其重要特征在于，通过分子水平的药效学实验，证实了这些黄连碱类生物碱衍生物分别显示出一定的或较显著的 *xbp*1 启动子转录激活效应，是在治疗溃疡性结肠炎方

面具有较高药用价值的化合物。此外，与底物比较，这些黄连碱类生物碱衍生物的溶解性能均增强，使它们在底物不易溶解的一些溶剂中均较易溶解或溶解性得到明显改善。

发明内容

本发明解决的技术问题是提供一种治疗溃疡性结肠炎的药物，即通式Ⅰ-Ⅴ所示的黄连碱类生物碱衍生物或其生理上可接受的盐。

为解决上述技术问题，本发明提供了如下技术方案：

本发明第一方面提供了一种通式Ⅰ-Ⅴ所示的黄连碱类生物碱衍生物或其生理上可接受的盐。

本发明第二方面提供了通式Ⅰ-Ⅴ所示的黄连碱类生物碱衍生物或其生理上可接受的盐的制备方法。

本发明第三方面提供了通式Ⅰ-Ⅴ所示的黄连碱类生物碱衍生物或其生理上可接受的盐的药物组合物。

本发明第四方面提供了通式Ⅰ-Ⅴ所示的黄连碱类生物碱衍生物或其生理上可接受的盐的治疗抗溃疡性结肠炎的用途。

本发明第一方面提供的通式Ⅰ-Ⅴ所示的黄连碱类生物碱衍生物或其生理上可接受的盐如下：

（1）如通式Ⅰ所示的黄连碱类生物碱季胺盐衍生物：

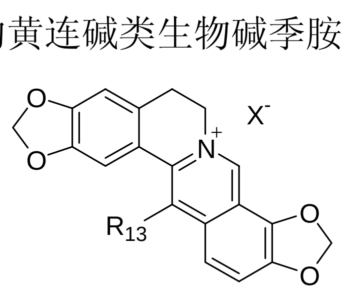

Ⅰ

其中，X^-为季铵盐负离子，优选的 X^-选自卤素负离子、硫酸根负离子等；

R_{13}选自CH_2OR_{13}'并且R_{13}'选自H或脂肪烃基，并且R_{13}'烃基通式为C_nH_{2n+1}，n选自1-13的整数，或R_{13}'脂肪烃基通式为C_mH_{2m-1}，m选自2-13的整数；

或R_{13}选自$COOR_{13}$'并且R_{13}'选自H或烃基，并且R_{13}'烃基通式为C_nH_{2n+1}，n选自1-5的整数；

或R_{13}选自CH_2COOR_{13}'并且R_{13}'选自H或烃基，并且R_{13}'烃基通式为C_nH_{2n+1}，n选自1-5的整数。

（2）如通式Ⅱ所示的黄连碱类生物碱季胺盐衍生物：

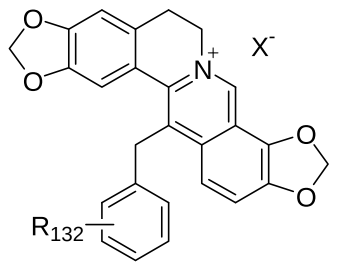

Ⅱ

其中，X^-为季铵盐负离子，优选的 X^-选自卤素负离子、硫酸根负离子等；

R_{132}选自H、三氟甲基、氰基、羟基、氨基、硝基、苯基、亚甲二氧基、1,2-亚乙二氧基、卤素、C1-C4的烷基，C1-C4的烷氧基，C1-C4的烷硫基，C1-C4的烷酰基、C1-C4的烷酰氧基；上述C1-C4的烷基，C1-C4的烷氧基，C1-C4的烷硫基，C1-C4的烷酰基、C1-C4的烷酰氧基中的烷基是直链或支链。

（3）如通式Ⅲ所示的二氢黄连碱类生物碱衍生物：

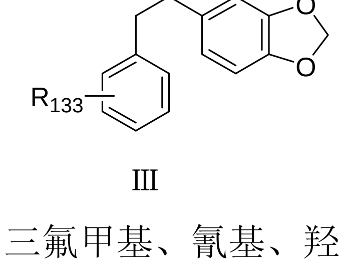

Ⅲ

其中，R_{133}选自H、三氟甲基、氰基、羟基、氨基、硝基、苯基、

亚甲二氧基、1,2-亚乙二氧基、卤素、C1-C4 的烷基，C1-C4 的烷氧基，C1-C4 的烷硫基，C1-C4 的烷酰基、C1-C4 的烷酰氧基；上述 C1-C4 的烷基，C1-C4 的烷氧基，C1-C4 的烷硫基，C1-C4 的烷酰基、C1-C4 的烷酰氧基中的烷基是直链或支链。

（4）如通式Ⅳ所示的四氢黄连碱类生物碱衍生物：

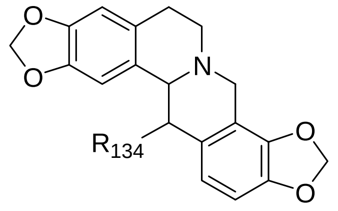

Ⅳ

其中， R_{134} 选自 CH_2COR_{13}' 并且 R_{13}' 选自 OH 或烃氧基，并且 R_{13}' 烃氧基通式为 OC_nH_{2n+1}，n 选自 1-13 的整数。

（5）如通式Ⅴ所示的四氢黄连碱类生物碱衍生物：

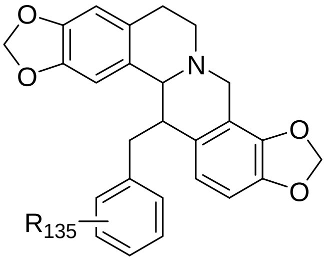

Ⅴ

其中，R_{135} 选自 H、三氟甲基、氰基、羟基、氨基、硝基、苯基、亚甲二氧基、1,2-亚乙二氧基、卤素、C1-C4 的烷基，C1-C4 的烷氧基，C1-C4 的烷硫基，C1-C4 的烷酰基、C1-C4 的烷酰氧基；上述 C1-C4 的烷基，C1-C4 的烷氧基，C1-C4 的烷硫基，C1-C4 的烷酰基、C1-C4 的烷酰氧基中的烷基是直链或支链。

本发明最优选的化合物选自如下式 **1-24** 所示化合物群组：

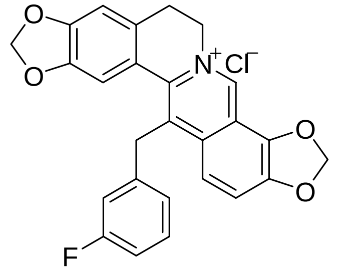

1

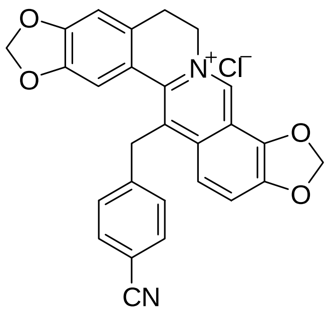

2

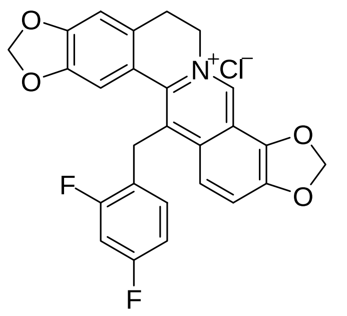

3

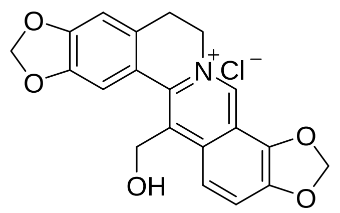

4

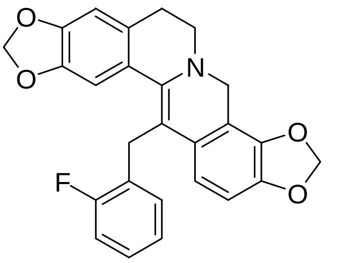

5

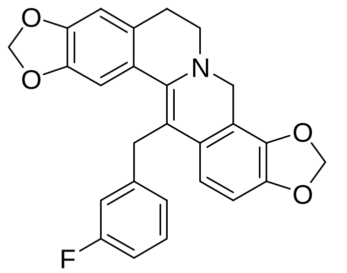

6

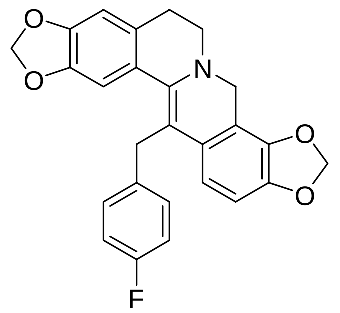

7

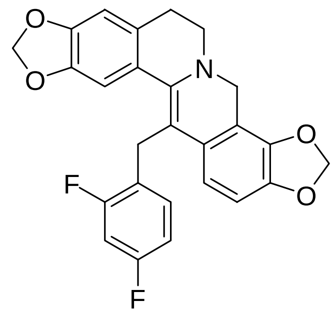

8

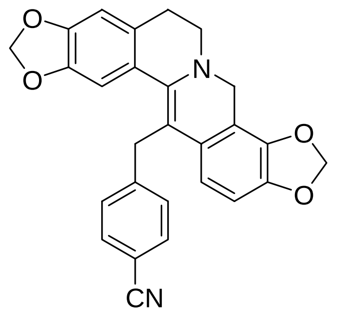

9

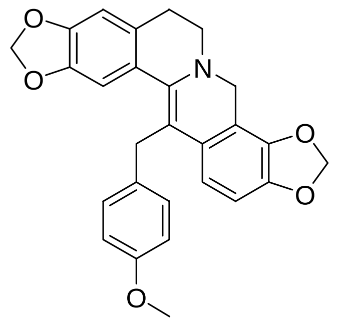

10

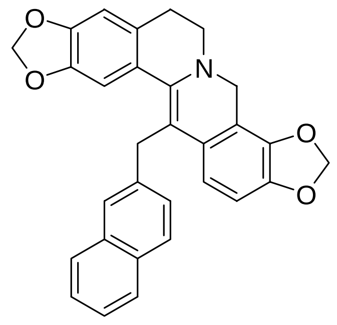

11

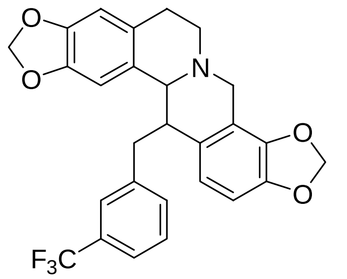

12

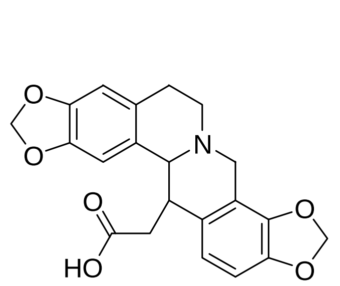

13

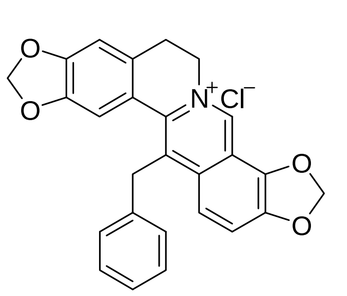

14

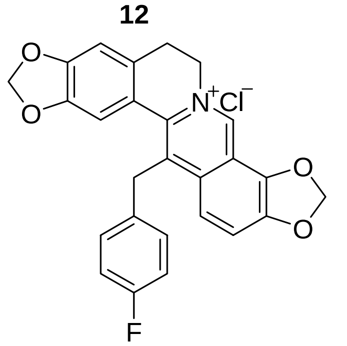

15

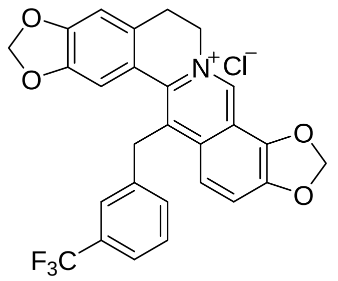

16

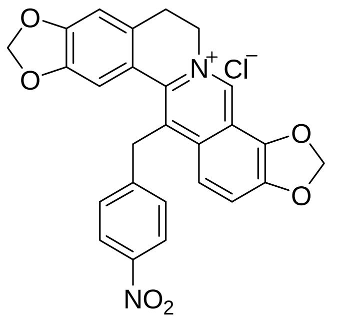

17

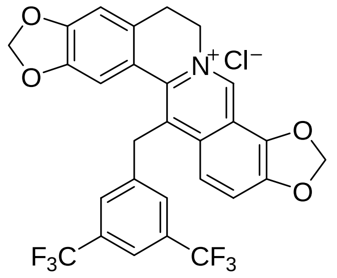

18

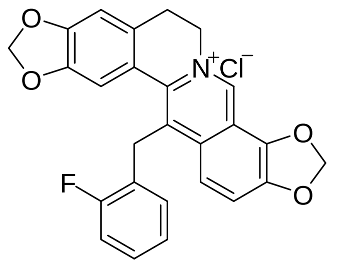

19

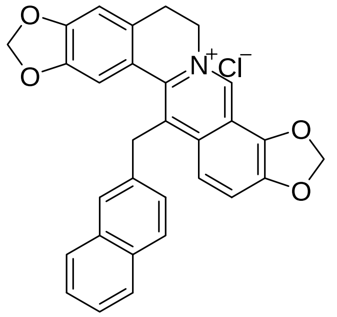

20

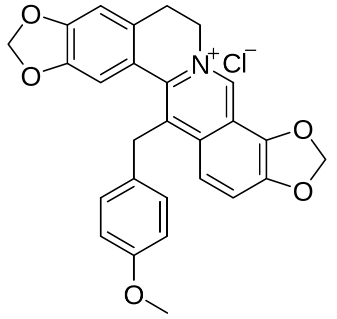

21

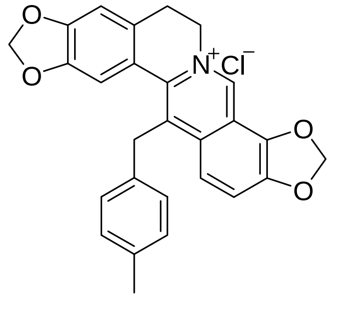

22

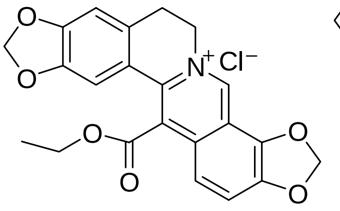

23

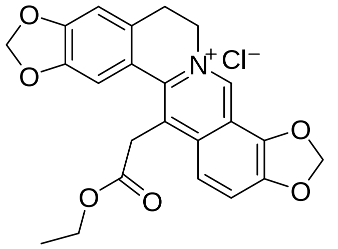

24

本发明第二方面提供了本发明化合物的制备方法：所述的黄连碱类生物碱衍生物或其生理上可接受的盐可通过如下的合成方法合成：

(1) 本发明的 13-羟甲基取代黄连碱季胺盐可通过如下的合成方法合成：

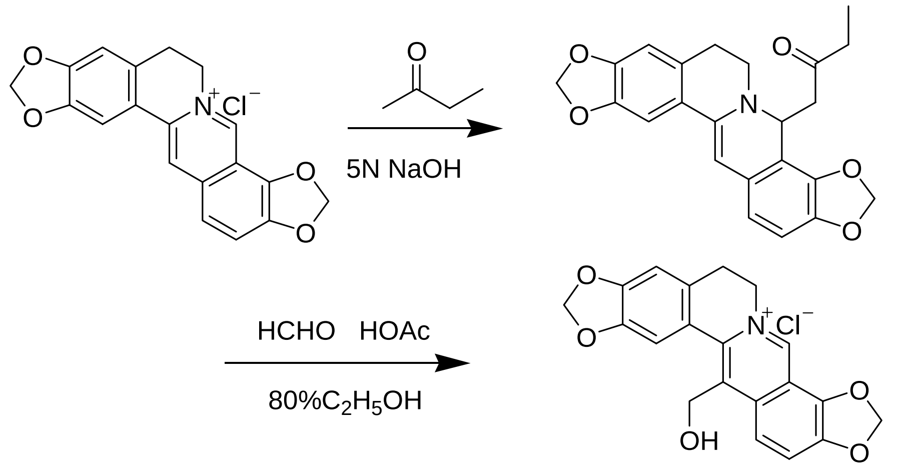

(2) 本发明的其它 13-取代黄连碱季铵盐类衍生物、13-取代二氢黄连碱类生物碱衍生物和 13-取代四氢黄连碱类生物碱衍生物可通过如下的合成方法合成（其中，X 代表卤素，R 代表苄基或取代苄基，或 R 代表羧酸酯基）：

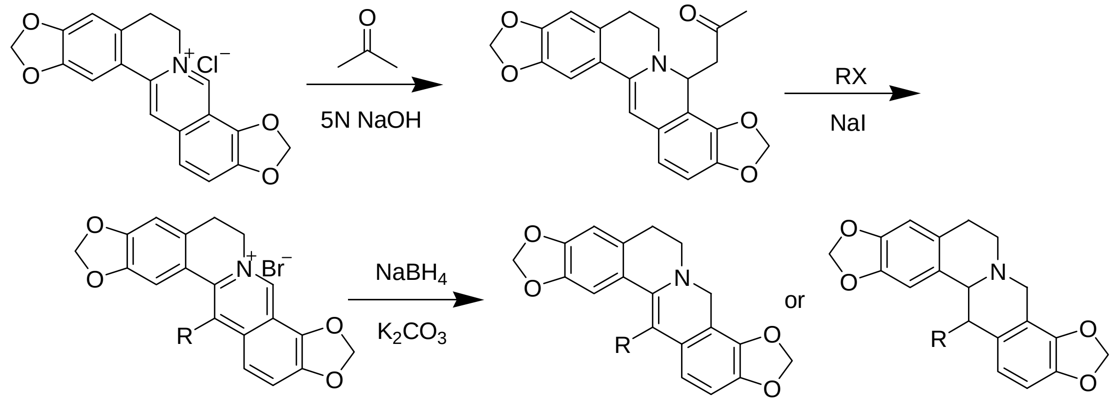

本发明第三方面还涉及以本发明化合物作为活性成份的药物组合物。该药物组合物可根据本领域公知的方法制备。可通过将本发明化合物与一种或多种药学上可接受的固体或液体赋形剂和/或辅剂结合，制成适于人或动物使用的任何剂型。本发明化合物在其药物组合物中的含量通常为 0.1-95 重量%。

本发明化合物或含有它的药物组合物可以单位剂量形式给药，给药途径可为肠道或非肠道，如口服、静脉注射、肌肉注射、皮下注射、鼻腔、口腔粘膜、眼、肺和呼吸道、皮肤、阴道、直肠等。

给药剂型可以是液体剂型、固体剂型或半固体剂型。液体剂型可以是溶液剂（包括真溶液和胶体溶液）、乳剂（包括 o/w 型、w/o 型和复乳）、混悬剂、注射剂（包括水针剂、粉针剂和输液）、滴眼剂、滴鼻剂、洗剂和搽剂等；固体剂型可以是片剂（包括普通片、肠溶片、含片、分散片、咀嚼片、泡腾片、口腔崩解片）、胶囊剂（包括硬胶囊、软胶囊、肠溶胶囊）、颗粒剂、散剂、微丸、滴丸、栓剂、膜剂、贴片、气（粉）雾剂、喷雾剂等；半固体剂型可以是软膏剂、凝胶剂、糊剂等。

本发明化合物可以制成普通制剂、也制成是缓释制剂、控释制剂、靶向制剂及各种微粒给药系统。

为了将本发明化合物制成片剂，可以广泛使用本领域公知的各种赋形剂，包括稀释剂、黏合剂、润湿剂、崩解剂、润滑剂、助流剂。稀释剂可以是淀粉、糊精、蔗糖、葡萄糖、乳糖、甘露醇、山梨醇、木糖醇、微晶纤维素、硫酸钙、磷酸氢钙、碳酸钙等；湿润剂可以是水、乙醇、异丙醇等；粘合剂可以是淀粉浆、糊精、糖浆、蜂蜜、葡萄糖溶液、微晶纤维素、阿拉伯胶浆、明胶浆、羧甲基纤维素钠、甲基纤维素、羟丙基甲基纤维素、乙基纤维素、丙烯酸树脂、卡波姆、聚乙烯吡咯烷酮、聚乙二醇等；崩解剂可以是干淀粉、微晶纤维素、低取代羟丙基纤维素、交联聚乙烯吡咯烷酮、交联羧甲基纤维素钠、羧甲基淀粉钠、碳酸氢钠与枸橼酸、聚氧乙烯山梨糖醇脂肪酸酯、十二烷基磺酸钠等；润滑剂和助流剂可以是滑石粉、二氧化硅、硬脂酸盐、酒石酸、液体石蜡、聚乙二醇等。

还可以将片剂进一步制成包衣片，例如糖包衣片、薄膜包衣片、肠溶包衣片，或双层片和多层片。

为了将给药单元制成胶囊剂，可以将有效成分本发明化合物与稀释剂、助流剂混合，将混合物直接置于硬胶囊或软胶囊中。也可将有

效成分本发明化合物先与稀释剂、黏合剂、崩解剂制成颗粒或微丸，再置于硬胶囊或软胶囊中。用于制备本发明化合物片剂的各稀释剂、黏合剂、润湿剂、崩解剂、助流剂品种也可用于制备本发明化合物的胶囊剂。

为将本发明化合物制成注射剂，可以用水、乙醇、异丙醇、丙二醇或它们的混合物作溶剂并加入适量本领域常用的增溶剂、助溶剂、pH 调剂剂、渗透压调节剂。增溶剂或助溶剂可以是泊洛沙姆、卵磷脂、羟丙基-β-环糊精等；pH 调剂剂可以是磷酸盐、醋酸盐、盐酸、氢氧化钠等；渗透压调节剂可以是氯化钠、甘露醇、葡萄糖、磷酸盐、醋酸盐等。如制备冻干粉针剂，还可加入甘露醇、葡萄糖等作为支撑剂。

此外，如需要，也可以向药物制剂中添加着色剂、防腐剂、香料、矫味剂或其它添加剂。

为达到用药目的，增强治疗效果，本发明的药物或药物组合物可用任何公知的给药方法给药。

本发明化合物药物组合物的给药剂量依照所要预防或治疗疾病的性质和严重程度，患者或动物的个体情况，给药途径和剂型等可以有大范围的变化。一般来讲，本发明化合物的每天的合适剂量范围为 0.001-150mg/Kg 体重,优选为 0.1-100mg/Kg 体重,更优选为 1-60mg/Kg 体重，最优选为 2-30mg/Kg 体重。上述剂量可以一个剂量单位或分成几个剂量单位给药，这取决于医生的临床经验以及包括运用其它治疗手段的给药方案。

本发明的化合物或组合物可单独服用，或与其他治疗药物或对症药物合并使用。当本发明的化合物与其它治疗药物存在协同作用时，应根据实际情况调整它的剂量。

本发明第四方面提供了化合物在制备治疗溃疡性结肠炎药物中的

应用。本发明所述黄连碱类生物碱衍生物或其生理上可接受的盐在分子水平的活性测试实验中分别显示出一定的或较显著的 *xbp*1 启动子转录激活效应，是在治疗溃疡性结肠炎方面具有较高药用价值的化合物。此外，与底物比较，这些黄连碱类生物碱衍生物的溶解性能均增强，使它们在底物不易溶解的一些溶剂中均较易溶解或溶解性得到明显改善。

附图说明

图 1　MTT 法检测各化合物对体外培养肠上皮细胞 IEC-6 的毒性结果。

图 2　本发明不同化合物对 *xbp*1 基因上游启动子的激活效应实验结果。

具体实施方式

实施例 1　化合物 1-24 的制备工艺及结构鉴定数据

化合物 **1** 的制备

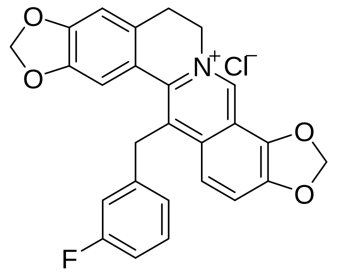

1

称取化合物 8-丙酮基取代二氢黄连碱(206 mg, 0.55 mmol)于反应瓶中，依次加入乙腈(6 mL)，3-氟溴苄(0.2 mL, 1.63 mmol)，NaI (82 mg, 0.55 mmol)，加完后回流反应 5 h，原料反应完全，将反应液浓缩，加入 2 N HCl (2 mL)，室温反应 1 h，用氯仿/甲醇(v/v= 10:1)萃取，有机相用无水 $MgSO_4$ 干燥，过滤，减压蒸除溶剂，粗品经硅胶柱层析[v/v= 30:1 (氯仿/甲醇)]纯化得黄色固体 160 mg，收率 57.1％。

^{1}H-NMR (DMSO-d_6) δ (ppm): 3.15 (br s, 2H, $NCH_2C\underline{H_2}$), 4.75 (s, 2H,

ArCH$_2$Ar), 4.80 (br s, 2H, NC$\underline{H}_2$CH$_2$), 6.08 (s, 2H, OCH$_2$O), 6.55 (s, 2H, OCH$_2$O), 6.92 (s, 1H, ArH), 7.00-7.16 (m, 3H, ArH), 7.16 (s, 1H, ArH), 7.36-7.43 (m, 1H, ArH), 7.59 (d, J = 8.7 Hz, 1H, ArH), 7.92 (d, J = 8.7 Hz, 1H, ArH), 10.10 (s, 1H, ArH).

化合物 **2** 的制备

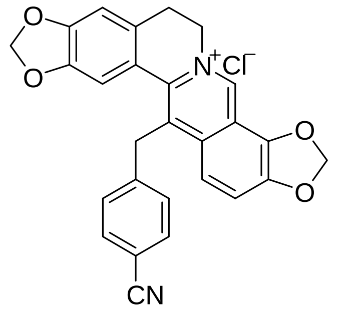

2

称取化合物 8-丙酮基取代二氢黄连碱(200 mg, 0.53 mmol)于反应瓶中，依次加入乙腈(6 mL)，对氰基溴苄(317 mg, 1.62 mmol)，NaI (80 mg, 0.53 mmol)，加完后回流反应 5 h，原料反应完全，将反应液浓缩，加入 2 N HCl (2 mL)，室温反应 1 h，用氯仿/甲醇(v/v= 10:1)萃取，有机相用无水 $MgSO_4$ 干燥，过滤，减压蒸除溶剂，粗品经硅胶柱层析[v/v= 30:1 (氯仿/甲醇)]纯化得黄色固体 142 mg，收率 52.0%。

^{1}H-NMR (DMSO-d_6) δ (ppm): 3.14 (t, J = 3.9 Hz, 2H, NCH$_2$C$\underline{H}_2$), 4.80 (br s, 2H, NC$\underline{H}_2$CH$_2$), 4.83 (s, 2H, ArCH$_2$), 6.08 (s, 2H, OCH$_2$O), 6.55 (s, 2H, OCH$_2$O), 6.83 (s, 1H, ArH), 7.15 (s, 1H, ArH), 7.37 (d, J = 5.7 Hz, 2H, ArH), 7.54 (d, J = 6.9 Hz, 1H, ArH), 7.82 (d, J = 6.3 Hz, 2H, ArH), 7.90 (d, J = 6.6 Hz, 1H, ArH), 10.10 (s, 1H, ArH).

化合物 **3** 的制备

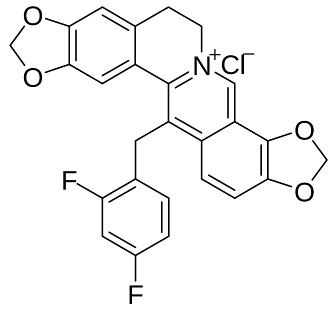

3

称取化合物 8-丙酮基取代二氢黄连碱(203 mg, 0.54 mmol)于反应瓶中，依次加入乙腈(6 mL)，2,4-二氟溴苄(0.2 mL, 1.56 mmol)，NaI (80 mg, 0.53 mmol)，加完后回流反应 5 h，原料反应完全，将反应液浓缩，加入 2 N HCl (2 mL)，室温反应 1 h，用氯仿/甲醇(v/v= 10:1)萃取，有机相用无水 $MgSO_4$ 干燥，过滤，减压蒸除溶剂，粗品经硅胶柱层析[v/v= 30:1 (氯仿/甲醇)]纯化得黄色固体 160 mg，收率 56.3％。

^{1}H-NMR (DMSO-d_6) δ (ppm): 3.13 (br s, 2H, $NCH_2C\underline{H_2}$), 4.62 (s, 2H, $ArCH_2$), 4.80 (br s, 2H, $NC\underline{H_2}CH_2$), 6.10 (s, 2H, OCH_2O), 6.56 (s, 2H, OCH_2O), 6.86 (s, 1H, ArH), 6.90-7.01 (m, 2H, ArH), 7.16 (s, 1H, ArH), 7.37-7.44 (m, 1H, ArH), 7.62 (d, *J* = 9.3 Hz, 1H, ArH), 7.94 (d, *J* = 8.7 Hz, 1H, ArH), 10.10 (s, 1H, ArH).

化合物 **4** 的制备

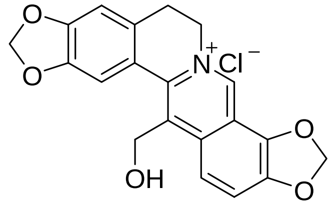

4

称取黄连碱(100 mg, 0.28 mmol)于反应瓶中，加入 5N NaOH (1.5 mL)，逐滴加入丁酮(0.2 mL, 2.23 mmol)，滴完后 60℃加热反应 3 h，停止反应，用氯仿/甲醇(v/v= 10:1)萃取，有机相水洗至中性，加入无水 $MgSO_4$ 干燥，过滤，减压蒸除溶剂，然后加入 80%乙醇(3 mL)，逐滴加入 HOAc (0.5 mL)，甲醛(0.7 mL, 7.03 mmol)，回流反应 3 h，原料反

应完全，将反应液浓缩，加入 2 N HCl (2 mL)，室温反应 1 h，然后用氯仿/甲醇(v/v= 10:1)萃取，有机相用无水 $MgSO_4$ 干燥，过滤，减压蒸除溶剂，粗品经硅胶柱层析[v/v= 20:1 (氯仿/甲醇)]纯化得黄色固体 23 mg，收率 21.2%。

^{1}H-NMR (DMSO-d_6) δ (ppm): 3.12 (br s, 2H, $NCH_2C\underline{H}_2$), 4.78 (s, 2H, $NC\underline{H}_2CH_2$), 4.81 (s, 2H, $C\underline{H}_2OH$), 6.18 (s, 2H, OCH_2O), 6.55 (s, 2H, OCH_2O), 7.16 (s, 1H, ArH), 7.52 (s, 1H, ArH), 7.98 (d, *J* = 9.0 Hz, 1H, ArH), 8.09 (d, *J* = 9.0 Hz, 1H, ArH), 10.06 (s, 1H, ArH).

化合物 **5** 的制备

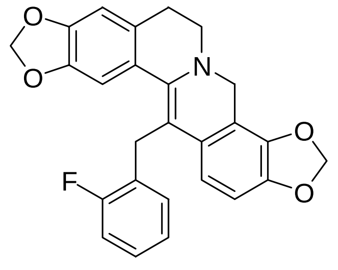

5

称取 13-(2-氟苄基)黄连碱(48 mg, 0.09 mmol)于反应瓶中，加入甲醇(4 mL)，K_2CO_3 (39 mg, 0.28 mmol)，称取 $NaBH_4$ (6 mg, 0.16 mmol) 溶解于 5%NaOH (0.5 mL)中，逐滴加入到反应瓶中，室温搅拌反应 2 h，原料反应完全，将反应液过滤，滤饼分别用水洗涤至中性，干燥得黄色固体 31 mg，收率 76.5%。

^{1}H-NMR (DMSO-d_6) δ (ppm): 2.74 (br s, 2H, $NCH_2C\underline{H}_2$), 3.07 (br s, 2H, $NC\underline{H}_2CH_2$), 3.95 (s, 2H, $ArCH_2Ar$), 4.29 (s, 2H, NCH_2Ar), 5.94 (s, 2H, OCH_2O), 5.99 (s, 2H, OCH_2O), 6.35 (d, *J* = 8.0 Hz, 1H, ArH), 6.59 (s, 1H, ArH), 6.63 (d, *J* = 8.0 Hz, 1H, ArH), 6.86 (s, 1H, ArH), 6.94-6.98 (m, 1H, ArH), 7.10-7.27 (m, 3H, ArH).

化合物 **6** 的制备

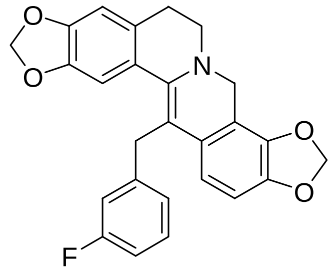

6

称取 13-(3-氟苄基)黄连碱(45 mg, 0.09 mmol)于反应瓶中，加入甲醇(4 mL)，加入 K_2CO_3 (38 mg, 0.27 mmol)，称取 $NaBH_4$ (5 mg, 0.13 mmol)溶解于 5%NaOH (0.5 mL)中，逐滴加入到反应瓶中，室温搅拌 2 h，原料反应完全，将反应液过滤，滤饼用水洗至中性，干燥得黄色固体 24 mg，收率 63.2％。

^{1}H-NMR (DMSO-d_6) δ (ppm): 2.75 (t, J = 4.8 Hz, 2H, $NCH_2C\underline{H_2}$), 3.09 (t, J = 4.8 Hz, 2H, $NC\underline{H_2}CH_2$), 4.05 (s, 2H, $ArCH_2Ar$), 4.29 (s, 2H, NCH_2Ar), 5.95 (s, 2H, OCH_2O), 6.00 (s, 2H, OCH_2O), 6.40 (d, J = 8.4 Hz, 1H, ArH), 6.64 (d, J = 8.4 Hz, 1H, ArH), 6.67 (s, 1H, ArH), 6.88 (s, 1H, ArH), 6.99-7.14 (m, 3H, ArH), 7.32-7.40 (m, 1H, ArH).

化合物 **7** 的制备

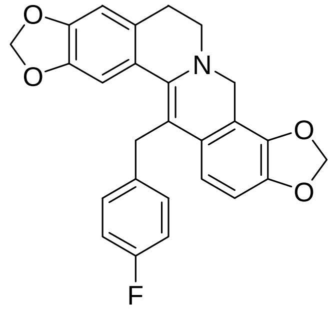

7

称取 13-(4-氟苄基)黄连碱(68 mg, 0.13 mmol)于反应瓶中，加入甲醇(4 mL)，加入 K_2CO_3 (54 mg, 0.39 mmol)，称取 $NaBH_4$ (7 mg, 0.19 mmol)溶解于 5%NaOH (0.5 mL)中，逐滴加入到反应瓶中，室温搅拌 2 h，原料反应完全，将反应液过滤，滤饼用水洗至中性，干燥得黄色固体 40 mg，收率 70.2％。

^{1}H-NMR (DMSO-d_6) δ (ppm): 2.73 (t, J = 4.5 Hz, 2H, NCH$_2$C$\underline{H_2}$), 3.07 (br s, 2H, NC$\underline{H_2}$CH$_2$), 3.99 (s, 2H, ArCH$_2$Ar), 4.27 (s, 2H, NCH$_2$Ar), 5.93 (s, 2H, OCH$_2$O), 5.99 (s, 2H, OCH$_2$O), 6.39 (d, J = 8.7 Hz, 1H, ArH), 6.62 (d, J = 8.1 Hz, 1H, ArH), 6.66 (s, 1H, ArH), 6.86 (s, 1H, ArH), 7.09-7.15 (m, 2H, ArH), 7.26-7.30 (m, 2H, ArH).

化合物 **8** 的制备

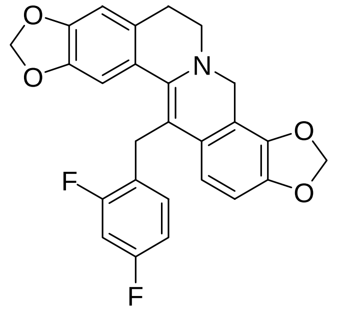

8

称取 13-(2,4-二氟苄基)黄连碱 (50 mg, 0.09 mmol)于反应瓶中，加入甲醇(3 mL)，加入 K_2CO_3 (37 mg, 0.27 mmol)，称取 $NaBH_4$ (5 mg, 0.13 mmol)溶解于 5%NaOH (0.5 mL)中，逐滴加入到反应瓶中，室温搅拌 2 h，原料反应完全，将反应液过滤，滤饼用水洗至中性，干燥得黄色固体 27 mg，收率 63.5％。

^{1}H-NMR (DMSO-d_6) δ (ppm): 2.75-2.77 (m, 2H, NCH$_2$C$\underline{H_2}$), 3.07-3.09 (m, 2H, NC$\underline{H_2}$CH$_2$), 3.93 (s, 2H, ArCH$_2$Ar), 4.30 (s, 2H, NCH$_2$Ar), 5.96 (s, 2H, OCH$_2$O), 6.01 (s, 2H, OCH$_2$O), 6.36 (d, J = 8.4 Hz, 1H, ArH), 6.57 (s, 1H, ArH), 6.65 (d, J = 8.4 Hz, 1H, ArH), 6.88 (s, 1H, ArH), 6.97-7.02 (m, 1H, ArH), 7.16-7.31 (m, 2H, ArH).

化合物 **9** 的制备

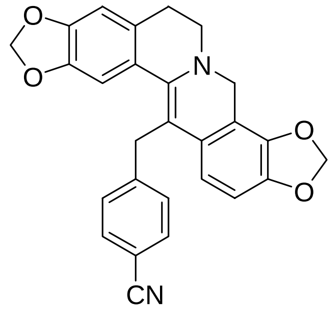

9

称取 13-(4-氰基苄基)黄连碱(64 mg, 0.12 mmol)于反应瓶中，加入甲醇(4 mL)，加入 K_2CO_3 (50 mg, 0.36 mmol)，称取 $NaBH_4$ (6 mg, 0.16 mmol)溶解于 5%NaOH (0.5 mL)中，逐滴加入到反应瓶中，室温搅拌 2 h，原料反应完全，将反应液过滤，滤饼用水洗至中性，干燥得黄色固体 39 mg，收率 72.0％。

^{1}H-NMR (DMSO-d_6) δ (ppm): 2.75 (br s, 2H, $NCH_2C\underline{H}_2$), 3.09 (br s, 2H, $NC\underline{H}_2CH_2$), 4.12 (s, 2H, $ArCH_2Ar$), 4.30 (s, 2H, NCH_2Ar), 5.95 (s, 2H, OCH_2O), 6.00 (s, 2H, OCH_2O), 6.35 (d, J = 8.4 Hz, 1H, ArH), 6.58 (s, 1H, ArH), 6.62 (d, J = 8.4 Hz, 1H, ArH), 6.88 (s, 1H, ArH), 7.47 (d, J = 8.1 Hz, 1H, ArH), 7.77 (d, J = 8.1 Hz, 1H, ArH).

化合物 **10** 的制备

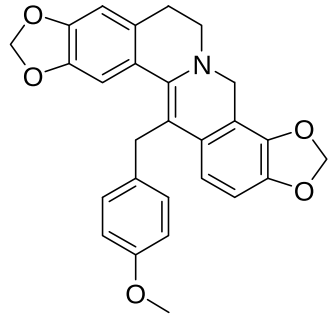

10

称取 13-(4-甲氧基苄基)黄连碱(52 mg, 0.10 mmol)于反应瓶中，加入甲醇(4 mL)，K_2CO_3 (41 mg, 0.30 mmol)，称取 $NaBH_4$ (6 mg, 0.16 mmol)溶解于 5%NaOH (0.5 mL)中，逐滴加入到反应瓶中，室温搅拌反应 2 h，原料反应完全，将反应液过滤，滤饼分别用水洗涤至中性，干燥得黄

色固体 35 mg，收率 79.5％。

^{1}H-NMR (DMSO-d_6) δ (ppm): 2.73 (br s, 2H, $NCH_2C\underline{H}_2$), 3.06 (br s, 2H, $NC\underline{H}_2CH_2$), 3.71 (s, 3H, OCH_3), 3.93 (s, 2H, $ArCH_2Ar$), 4.25 (s, 2H, NCH_2Ar), 5.92 (s, 2H, OCH_2O), 5.98 (s, 2H, OCH_2O), 6.40 (d, J = 8.0 Hz, 1H, ArH), 6.61 (d, J = 8.0 Hz, 1H, ArH), 6.73 (s, 1H, ArH), 6.85 (s, 1H, ArH), 6.86 (d, J = 8.0 Hz, 1H, ArH), 7.16 (d, J = 8.0 Hz, 1H, ArH).

化合物 **11** 的制备

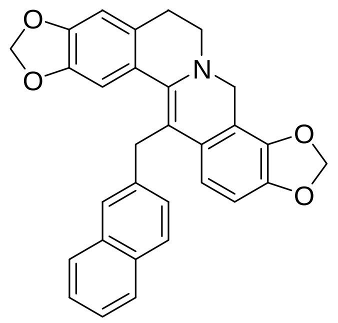

11

称取 13-(2-萘甲基)黄连碱(56 mg, 0.10 mmol)于反应瓶中，加入甲醇(4 mL)，K_2CO_3 (43 mg, 0.31 mmol)，称取 $NaBH_4$ (6 mg, 0.16 mmol)溶解于 5%NaOH (0.5 mL)中，逐滴加入到反应瓶中，室温搅拌反应 2 h，原料反应完全，将反应液过滤，滤饼分别用水洗涤至中性，干燥得黄色固体 39 mg，收率 81.6％。

^{1}H-NMR (DMSO-d_6) δ (ppm): 2.76 (br s, 2H, $NCH_2C\underline{H}_2$), 3.11 (br s, 2H, $NC\underline{H}_2CH_2$), 4.17 (s, 2H, $ArCH_2Ar$), 4.34 (s, 2H, NCH_2Ar), 5.88 (s, 2H, OCH_2O), 5.99 (s, 2H, OCH_2O), 6.42 (d, J = 8.0 Hz, 1H, ArH), 6.57 (d, J = 8.0 Hz, 1H, ArH), 6.76 (s, 1H, ArH), 6.86 (s, 1H, ArH), 7.45-7.49 (m, 3H, ArH), 7.71 (s, 1H, ArH), 7.79-7.80 (m, 1H, ArH), 7.86-7.88 (m, 2H, ArH).

化合物 **12** 的制备

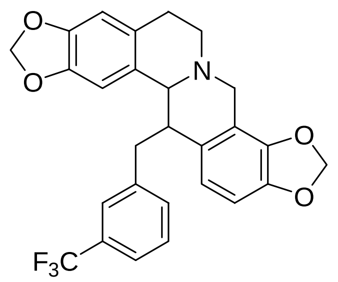

12

称取 13-(3-三氟苄基)黄连碱(51 mg, 0.09 mmol)于反应瓶中，加入甲醇(4 mL)，加入 K_2CO_3 (38 mg, 0.27 mmol)，称取 $NaBH_4$ (7 mg, 0.19 mmol)溶解于 5%NaOH (0.5 mL)中，逐滴加入到反应瓶中，室温搅拌 3 h，原料反应完全，将反应液过滤，滤饼用水洗至中性，干燥得黄色固体 32 mg，收率 72.7％。

^{1}H-NMR (DMSO-d_6) δ (ppm): 2.44-2.51 (m, 1H), 2.56-2.65 (m, 2H), 2.70-2.76 (m, 1H), 2.92-3.02 (m, 1H), 3.09-3.13 (m, 1H), 3.45 (d, J = 15.6 Hz, 1H), 3.55 (br s, 1H), 3.72 (br s, 1H), 4.05 (d, J = 15.6 Hz, 1H), 5.89 (s, 1H), 5.93-6.00 (m, 4H), 6.55 (d, J = 8.1 Hz, 1H, ArH), 6.66 (s, 1H, ArH), 6.95 (s, 1H, ArH), 7.05 (s, 1H, ArH), 7.18 (d, J = 6.9 Hz, 1H, ArH), 7.35-7.45 (m, 2H, ArH).

化合物 **13** 的制备

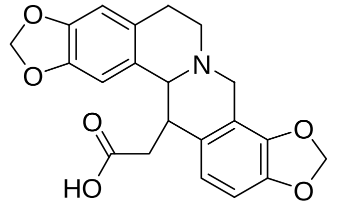

13

称取 13-黄连碱乙酸乙酯(46 mg, 0.10 mmol)于反应瓶中，加入甲醇(4 mL)，加入 K_2CO_3 (43 mg, 0.31 mmol)，称取 $NaBH_4$ (6 mg, 0.16 mmol)溶解于 5%NaOH (0.3 mL)中，逐滴加入到反应瓶中，室温搅拌 3 h，原料反应完全，用 2 N HCl 调节 PH=5，将反应液过滤，滤饼用水洗至中性，干燥得浅黄色固体 25 mg，收率 62.5％。

^{1}H-NMR (DMSO-d_6) δ (ppm): 2.13 (d, J = 15.2 Hz, 1H), 2.20-2.29 (m, 1H), 2.40-2.45 (m, 1H), 2.55 (d, J = 15.2 Hz, 1H), 2.83-2.90 (m, 1H), 3.04-3.06 (m, 1H), 3.42 (d, J = 15.2 Hz, 1H), 3.63-3.64 (m, 1H), 3.67 (s, 1H), 3.98 (d, J = 15.2 Hz, 1H), 5.94-6.01 (m, 4H, OCH_2O), 6.59 (d, J = 8.0 Hz, 1H, ArH), 6.68 (s, 1H, ArH), 6.74 (d, J = 8.0 Hz, 1H, ArH), 6.88 (s, 1H, ArH).

化合物 **14** 的制备

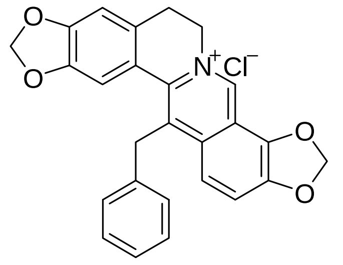

14

称取化合物 8-丙酮基取代二氢黄连碱(105 mg, 0.28 mmol)于反应瓶中，依次加入乙腈(4 mL)，溴苄(0.1 mL, 0.84 mmol)，NaI (42 mg, 0.28 mmol)，加完后回流反应 5 h，原料反应完全，将反应液浓缩，加入 2 N HCl (1.0 mL)，室温反应 1 h，然后用氯仿/甲醇(v/v= 10:1)萃取，有机相用无水 $MgSO_4$ 干燥，过滤，减压蒸除溶剂，粗品经硅胶柱层析[v/v= 30:1 (氯仿/甲醇)]纯化得黄色固体 85 mg，收率 62.0%。

^{1}H-NMR (DMSO-d_6) δ (ppm): 3.14 (br s, 2H, $NCH_2C\underline{H_2}$), 4.73 (s, 2H, $ArCH_2$), 4.81 (br s, 2H, $NC\underline{H_2}CH_2$), 6.07 (s, 2H, OCH_2O), 6.55 (s, 2H, OCH_2O), 6.96 (s, 1H, ArH), 7.15 (s, 1H, ArH), 7.18-7.36 (m, 5H, ArH), 7.59 (d, J = 8.7 Hz, 1H, ArH), 7.92 (d, J = 8.7 Hz, 1H, ArH), 10.08 (s, 1H, ArH).

化合物 **15** 的制备

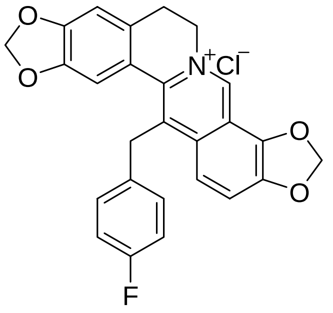

15

称取化合物 8-丙酮基取代二氢黄连碱(203 mg, 0.54 mmol)于反应瓶中，依次加入乙腈(6 mL)，对氟溴苄(0.2 mL, 1.45 mmol)，NaI (80 mg, 0.53 mmol)，加完后回流反应 5 h，原料反应完全，将反应液浓缩，加入 2 N HCl (1.0 mL)，室温反应 1 h，用氯仿/甲醇(v/v= 10:1)萃取，有机相用无水 $MgSO_4$ 干燥，过滤，减压蒸除溶剂，粗品经硅胶柱层析[v/v= 30:1 (氯仿/甲醇)]纯化得黄色固体 163 mg，收率 59.4％。

^{1}H-NMR ($CDCl_3$) δ (ppm): 3.22 (br s, 2H, $NCH_2C\underline{H_2}$), 4.63 (s, 2H, $ArCH_2$), 5.29 (br s, 2H, $NC\underline{H_2}CH_2$), 6.00 (s, 2H, OCH_2O), 6.44 (s, 2H, OCH_2O), 6.88 (d, *J* = 4.5 Hz, 2H, ArH), 7.03-7.07 (m, 4H, ArH), 7.41 (d, *J* = 6.9 Hz, 1H, ArH), 7.53 (d, *J* = 6.6 Hz, 1H, ArH), 10.88 (s, 1H, ArH).

化合物 **16** 的制备

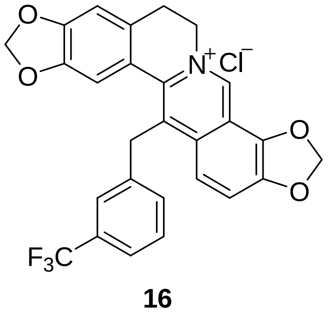

16

称取化合物 8-丙酮基取代二氢黄连碱(200 mg, 0.53 mmol)于反应瓶中，依次加入乙腈(6 mL)，间三氟甲基溴苄(0.25 mL, 1.64 mmol)，NaI (80 mg, 0.53 mmol)，加完后回流反应 5 h，原料反应完全，将反应液浓缩，加入 2 N HCl (1.0 mL)，室温反应 1 h，用氯仿/甲醇(v/v= 10:1)萃取，有机相用无水 $MgSO_4$ 干燥，过滤，减压蒸除溶剂，粗品经硅胶

柱层析[v/v= 30:1 (氯仿/甲醇)]纯化得黄色固体 186 mg，收率 62.8%。

^{1}H-NMR ($CDCl_3$) δ (ppm): 3.24 (t, *J* = 2.4 Hz, 2H, $NCH_2C\underline{H}_2$), 4.73 (s, 2H, $ArCH_2$), 5.33 (br s, 2H, $NC\underline{H}_2CH_2$), 6.01 (s, 2H, OCH_2O), 6.47 (s, 2H, OCH_2O), 6.80 (s, 1H, ArH), 6.89 (s, 1H, ArH), 7.36 (d, *J* = 8.7 Hz, 1H, ArH), 7.44-7.61 (m, 5H, ArH), 11.00 (s, 1H, ArH).

化合物 **17** 的制备

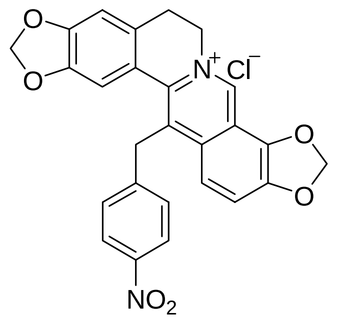

17

称取化合物 8-丙酮基取代二氢黄连碱(200 mg, 0.53 mmol)于反应瓶中，依次加入乙腈(6 mL)，对硝基溴苄(347 mg, 1.61 mmol)，NaI (80 mg, 0.53 mmol)，加完后回流反应 5 h，原料反应完全，将反应液浓缩，加入 2 N HCl (1.0 mL)，室温反应 1 h，用氯仿/甲醇(v/v= 10:1)萃取，有机相用无水 $MgSO_4$ 干燥，过滤，减压蒸除溶剂，粗品经硅胶柱层析[v/v= 30:1 (氯仿/甲醇)]纯化得黄色固体 53 mg，收率 18.7%。

^{1}H-NMR (DMSO-d_6) δ (ppm): 3.15 (t, *J* = 3.9 Hz, 2H, $NCH_2C\underline{H}_2$), 4.80 (br s, 2H, $NC\underline{H}_2CH_2$), 4.88 (s, 2H, $ArCH_2$), 6.07 (s, 2H, OCH_2O), 6.55 (s, 2H, OCH_2O), 6.84 (s, 1H, ArH), 7.16 (s, 1H, ArH), 7.44 (d, *J* = 6.0 Hz, 2H, ArH), 7.55 (d, *J* = 6.6 Hz, 1H, ArH), 7.90 (d, *J* = 6.9 Hz, 1H, ArH), 8.19 (d, *J* = 6.0 Hz, 2H, ArH), 10.10 (s, 1H, ArH).

化合物 **18** 的制备

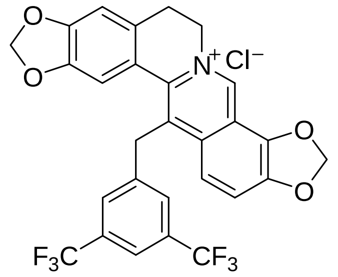

18

称取化合物 8-丙酮基取代二氢黄连碱(203 mg, 0.54 mmol)于反应瓶中，依次加入乙腈(6 mL)，3, 5-双三氟甲基氯苄(425 mg, 1.62 mmol)，NaI (80 mg, 0.53 mmol)，加完后回流反应 5 h，原料反应完全，将反应液浓缩，用氯仿/甲醇(v/v= 10:1)萃取，有机相用无水 $MgSO_4$ 干燥，过滤，减压蒸除溶剂，粗品经硅胶柱层析[v/v= 30:1 (氯仿/甲醇)]纯化得黄色固体 58 mg，收率 18.5％。

^{1}H-NMR (DMSO-d_6) δ (ppm): 3.10 (t, J = 3.9 Hz, 2H, $NCH_2C\underline{H_2}$), 4.78 (br s, 2H, $NC\underline{H_2}CH_2$), 4.99 (s, 2H, $ArCH_2$), 6.05 (s, 2H, OCH_2O), 6.56 (s, 2H, OCH_2O), 6.88 (s, 1H, ArH), 7.10 (s, 1H, ArH), 7.69 (d, J = 6.6 Hz, 1H, ArH), 7.83 (s, 2H, ArH), 7.94 (d, J = 6.9 Hz, 1H, ArH), 7.98 (s, 1H, ArH), 10.11 (s, 1H, ArH).

化合物 **19** 的制备

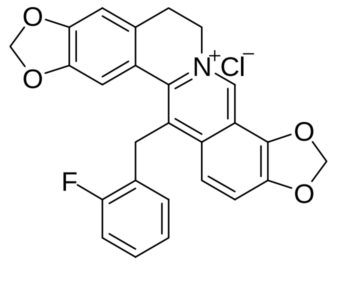

19

称取化合物 8-丙酮基取代二氢黄连碱(205 mg, 0.54 mmol)于反应瓶中，依次加入乙腈(6 mL)，邻氟溴苄(0.2 mL, 1.66 mmol)，NaI (82 mg, 0.55 mmol)，加完后回流反应 5 h，原料反应完全，将反应液浓缩，加入 2 N HCl (1.0 mL)，室温反应 1 h，用氯仿/甲醇(v/v= 10:1)萃取，有机

相用无水 $MgSO_4$ 干燥，过滤，减压蒸除溶剂，粗品经硅胶柱层析[v/v= 30:1 (氯仿/甲醇)]纯化得黄色固体 110 mg，收率 40.1％。

^{1}H-NMR (DMSO-d_6) δ (ppm): 3.14 (br s, 2H, $NCH_2C\underline{H_2}$), 4.65 (s, 2H, $ArCH_2$), 4.80 (br s, 2H, $NC\underline{H_2}CH_2$), 6.08 (s, 2H, OCH_2O), 6.56 (s, 2H, OCH_2O), 6.82-6.84 (m, 1H, ArH), 6.86 (s, 1H, ArH), 7.05-7.10 (m, 1H, ArH), 7.16 (s, 1H, ArH), 7.33-7.37 (m, 2H, ArH), 7.61 (d, J = 5.7 Hz, 1H, ArH), 7.93 (d, J = 5.7 Hz, 1H, ArH), 10.11 (s, 1H, ArH).

化合物 **20** 的制备

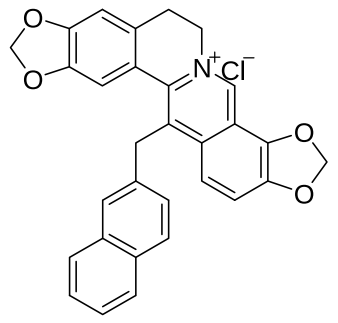

20

称取化合物 8-丙酮基取代二氢黄连碱(205 mg, 0.54 mmol)于反应瓶中，依次加入乙腈(6 mL)，2-溴甲基萘(362 mg, 1.64 mmol)，NaI (80 mg, 0.53 mmol)，加完后回流反应 5 h，原料反应完全，将反应液浓缩，加入 2 N HCl (1.0 mL)，室温反应 1 h，用氯仿/甲醇(v/v= 10:1)萃取，有机相用无水 $MgSO_4$ 干燥，过滤，减压蒸除溶剂，粗品经硅胶柱层析[v/v= 30:1 (氯仿/甲醇)]纯化得黄色固体 192 mg，收率 65.8％。

^{1}H-NMR (DMSO-d_6) δ (ppm): 3.17 (br s, 2H, $NCH_2C\underline{H_2}$), 4.87 (br s, 3H, $NC\underline{H_2}CH_2$, $ArCH_2$), 6.02 (s, 2H, OCH_2O), 6.56 (s, 2H, OCH_2O), 6.99 (s, 1H, ArH), 7.16 (s, 1H, ArH), 7.44-7.50 (m, 4H, ArH), 7.61-7.70 (m, 2H, ArH), 7.86-7.98 (m, 3H, ArH), 10.14 (s, 1H, ArH).

化合物 **21** 的制备

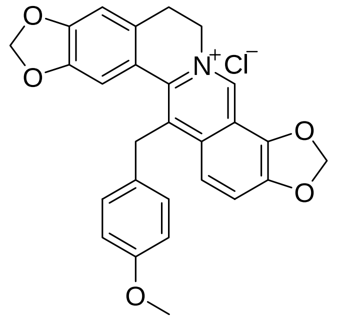

21

称取化合物 8-丙酮基取代二氢黄连碱(203 mg, 0.54 mmol)于反应瓶中，依次加入乙腈(6 mL)，对甲氧基溴苄(0.25 mL, 1.71 mmol)，NaI (83 mg, 0.55 mmol)，加完后回流反应 5 h，原料反应完全，将反应液浓缩，加入 2 N HCl (1.0 mL)，室温反应 1 h，用氯仿/甲醇(v/v= 10:1)萃取，有机相用无水 $MgSO_4$ 干燥，过滤，减压蒸除溶剂，粗品经硅胶柱层析[v/v= 30:1 (氯仿/甲醇)]纯化得黄色固体 248 mg，收率 86.7％。

^{1}H-NMR (DMSO-d_6) δ (ppm): 3.14 (br s, 2H, $NCH_2C\underline{H_2}$), 4.63 (s, 2H, $ArCH_2$), 4.81 (br s, 2H, $NC\underline{H_2}CH_2$), 6.07 (s, 2H, OCH_2O), 6.54 (s, 2H, OCH_2O), 6.91 (d, J = 8.4 Hz, 2H, ArH), 6.99 (s, 1H, ArH), 7.07 (d, J = 8.4 Hz, 2H, ArH), 7.15 (s, 1H, ArH), 7.59 (d, J = 9.0 Hz, 1H, ArH), 7.92 (d, J = 9.3 Hz, 1H, ArH), 10.07 (s, 1H, ArH).

化合物 **22** 的制备

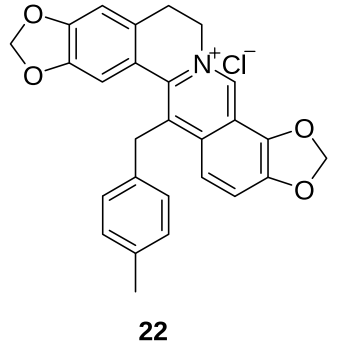

22

称取化合物 8-丙酮基取代二氢黄连碱(208 mg, 0.55 mmol)于反应瓶中，依次加入乙腈(6 mL)，对甲基溴苄(308 mg, 1.66 mmol)，NaI (83 mg, 0.55 mmol)，加完后回流反应 5 h，原料反应完全，将反应液浓缩，

加入 2 N HCl (1.0 mL)，室温反应 1 h，用氯仿/甲醇(v/v= 10:1)萃取，有机相用无水 $MgSO_4$ 干燥，过滤，减压蒸除溶剂，粗品经硅胶柱层析[v/v= 30:1 (氯仿/甲醇)]纯化得黄色固体 222 mg，收率 80.0％。

^{1}H-NMR (DMSO-d_6) δ (ppm): 2.29 (s, 3H, $ArCH_3$), 3.15 (br s, 2H, $NCH_2C\underline{H}_2$), 4.67 (s, 2H, $ArCH_2$), 4.82 (br s, 2H, $NC\underline{H}_2CH_2$), 6.08 (s, 2H, OCH_2O), 6.56 (s, 2H, OCH_2O), 6.97 (s, 1H, ArH), 7.07 (d, J = 7.5 Hz, 2H, ArH), 7.16 (s, 1H, ArH), 7.17 (d, J = 7.2 Hz, 2H, ArH), 7.57 (d, J = 9.0 Hz, 1H, ArH), 7.92 (d, J = 9.0 Hz, 1H, ArH), 10.09 (s, 1H, ArH).

化合物 **23** 的制备

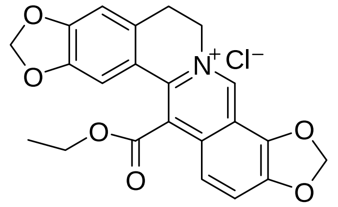

23

称取化合物 8-丙酮基取代二氢黄连碱(200 mg, 0.53 mmol)于反应瓶中，依次加入乙腈(6 mL)，氯甲酸乙酯(0.2 mL, 2.09 mmol)，NaI (80 mg, 0.53 mmol)，加完后回流反应 8 h，原料反应完全，将反应液浓缩，加入 2 N HCl (1.5 mL)，室温反应 1 h，然后用氯仿/甲醇(v/v= 10:1)萃取，有机相用无水 $MgSO_4$ 干燥，过滤，减压蒸除溶剂，粗品经硅胶柱层析[v/v= 30:1 (氯仿/甲醇)]纯化得黄色固体 40 mg，收率 17.6％。

^{1}H-NMR (DMSO-d_6) δ (ppm): 1.30 (t, J = 6.9 Hz, 3H, $CH_2C\underline{H}_3$), 3.16 (br s, 2H, $NCH_2C\underline{H}_2$), 4.51 (q, J = 6.9 Hz, 2H, $C\underline{H}_2CH_3$), 4.81 (br s, 2H, $NC\underline{H}_2CH_2$), 6.18 (s, 2H, OCH_2O), 6.60 (s, 2H, OCH_2O), 7.10 (s, 1H, ArH), 7.17 (s, 1H, ArH), 7.66 (d, J = 9.3 Hz, 1H, ArH), 8.08 (d, J = 9.0 Hz, 1H, ArH), 10.17 (s, 1H, ArH).

化合物 **24** 的制备

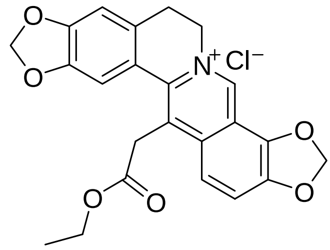

24

称取化合物 8-丙酮基取代二氢黄连碱(230 mg, 0.61 mmol)于反应瓶中，依次加入乙腈(6 mL)，溴乙酸乙酯(0.2 mL, 1.81 mmol)，NaI (92 mg, 0.61 mmol)，加完后回流反应 8 h，原料反应完全，将反应液浓缩，加入 2 N HCl (1.0 mL)，室温反应 1 h，然后用氯仿/甲醇(v/v= 10:1)萃取，有机相用无水 $MgSO_4$ 干燥，过滤，减压蒸除溶剂，粗品经硅胶柱层析[v/v= 30:1 (氯仿/甲醇)]纯化得黄色固体 120 mg，收率 44.4％。

^{1}H-NMR (DMSO-d_6) δ (ppm): 1.24 (t, J = 7.2 Hz, 3H, $CH_2C\underline{H}_3$), 3.09 (br s, 2H, $NCH_2C\underline{H}_2$), 4.22 (q, J = 7.2 Hz, 2H, $C\underline{H}_2CH_3$), 4.42 (s, 2H, $ArCH_2$), 4.77 (br s, 2H, $NC\underline{H}_2CH_2$), 6.18 (s, 2H, OCH_2O), 6.56 (s, 2H, OCH_2O), 7.15 (s, 1H, ArH), 7.17 (s, 1H, ArH), 7.80 (d, J = 8.7 Hz, 1H, ArH), 8.07 (d, J = 8.7 Hz, 1H, ArH), 10.04 (s, 1H, ArH).

药理实验

实验例 1

化合物的抗溃疡性结肠炎生物学研究实施实例

1、化合物的细胞毒性检测

（1）实验方法：将体外培养生长至 90%汇合状态的 IEC-6 肠上皮细胞以 0.25%胰酶/0.1% EDTA 消化并接种于 96 孔细胞培养板，每孔细胞数为 2×103。培养次日去除原培养基，每孔加入含 1×10^{-5}mol/L 的待测化合物工作液继续培养。于 IEC-6 细胞与待测药物共培养后的 0h，24h 和 72h 通过 MTT 法检测待测药物对细胞的毒性(n=5)。

（2）结果：在实验测定的时间范围内，1×10^{-5}mol/L 本发明所涉

及的系列化合物对于肠上皮细胞 IEC-6 均无显著的细胞毒性，统计学上检测无显著性差异（见图 1）。

（3）结论：本发明涉及系列新的黄连碱类生物碱衍生物或其生理上可接受的盐适于在 IEC-6 细胞模型上用于下游实验的筛选。

本发明所涉及系列新的黄连碱类生物碱衍生物或其生理上可接受的盐在浓度为 1uM 时对肠上皮 IEC-6 细胞毒性检测结果如图 1 所示。实验表明：化合物 **1-13** 在该浓度下与 IEC-6 细胞共孵育 24h 后无明显细胞毒作用，共孵育 3d 后检测结果与 24h 一致（此处未展示），均未表现出明显的细胞毒效应。

2、13 个无明显 IEC-6 细胞毒的化合物对 pGL3-p*xbp*1 的转录激活效应。

（1）实验方法：将处于生长旺盛期的 IEC-6 细胞接种于 48 孔板中，每孔细胞数为 5×104，使细胞在孔内均匀分散，放置于 37℃、5%CO_2 加湿细胞培养箱培养。待细胞汇片至 70%-80%，对细胞进行相应质粒的转染（0.6μg/孔），4h 后加入 1×10^{-5}mol/L 的各化合物与转染细胞共孵育(n=3)。待共培养 36h-48h 后收样，利用双荧光素酶报告基因检测试剂盒(Promega，USA)对实验样品进行荧光素酶活性检测。

（2）结果：以无转染质粒细胞为对照组 1，以转染 pGL-*xbp*1 不加药细胞组为对照组 2，经统计学分析结果显示有 13 个化合物对 *xbp*1 基因上游启动子具有激活效应。

（3）结论：本发明所涉及系列新的黄连碱类生物碱衍生物或其生理上可接受的盐对 *xbp*1 基因的表达具有转录激活效应。

实验结果见图 2。

不同化合物对 *xbp*1 基因启动子具有一定的转录激活效应。图中 con1 为本底对照，con2 为 pGL3 空载体对照。结果表明，该类新结构化合物不同程度上能激活 *xbp*1 分子的转录，具有一定的转录激活效应

说 明 书 附 图

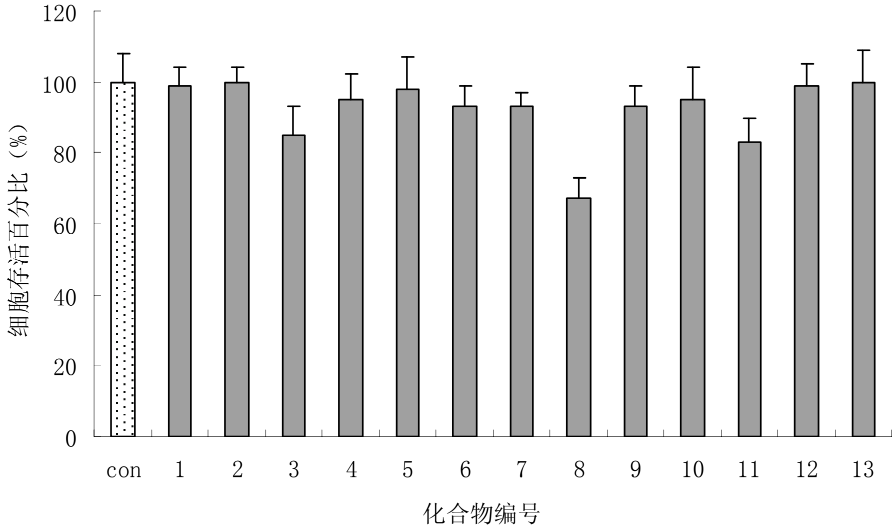

120
100
80
60
40
20
0
细胞存活百分比（%）
con 1 2 3 4 5 6 7 8 9 10 11 12 13
化合物编号

图 1

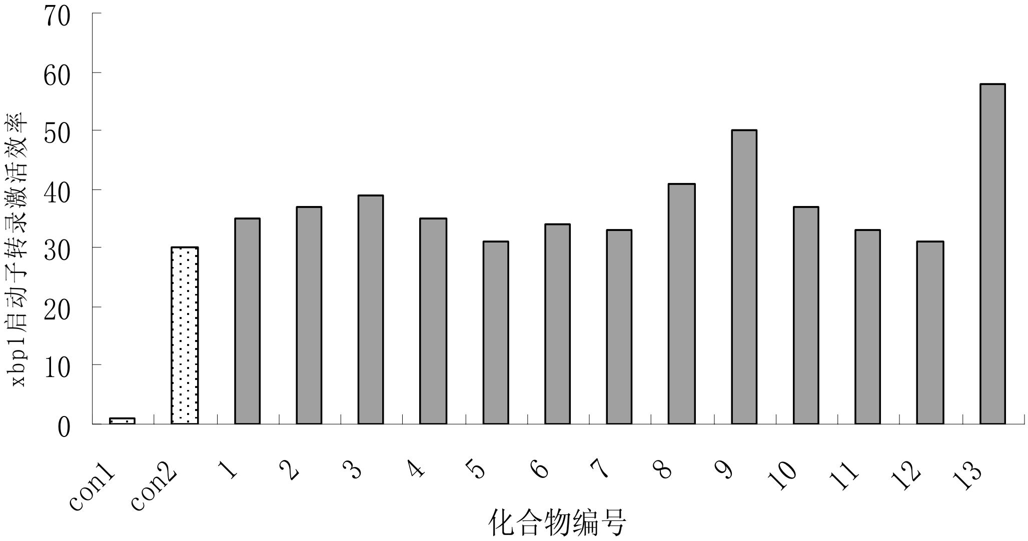

70
60
50
40
30
20
10
0
xbp1启动子转录激活效率
con1 con2 1 2 3 4 5 6 7 8 9 10 11 12 13
化合物编号

图 2

专 利 代 理 委 托 书

请按照“注意事项”正确填写本表各栏

根据专利法第 19条的规定

委　托 北京三高永信知识产权代理有限责任公司 机构代码（11138）

1. 代为办理名称为 黄连碱类生物碱衍生物及其抗溃疡性结肠炎的用途 的发明创造

申请或专利（申请号或专利号为__________）以及在专利权有效期内的全部专利事务。

☐委托人声明委托上述专利代理机构办理专利费用减缓手续。

2. 代为办理名称为__________

专利号为__________的实用新型专利检索报告。

3. 代为办理名称为__________

专利号为__________的专利权评价报告。

4. 其他

专利代理机构接收上述委托并指定专利代理人

【代理人姓名】 何文彬

【代理人姓名】

办理此项委托。

委托人（单位或个人）　中国医学科学院药物研究所　（盖章或签字）

____

被委托人（专利代理机构）　北京三高永信知识产权代理有限责任公司

（盖章）

____

2013年 5月 28日

专 利 代 理 委 托 书

【此处插入专利代理委托书扫描文件】
